# Dermatoglyphics from All Chinese Ethnic Groups Reveal Geographic Patterning

**DOI:** 10.1371/journal.pone.0008783

**Published:** 2010-01-20

**Authors:** Hai-Guo Zhang, Yao-Fong Chen, Ming Ding, Li Jin, D. Troy Case, Yun-Ping Jiao, Xian-Ping Wang, Chong-Xian Bai, Gang Jin, Jiang-Ming Yang, Han Wang, Jian-Bing Yuan, Wei Huang, Zhu-Gang Wang, Ren-Biao Chen

**Affiliations:** 1 Department of Medical Genetics, Shanghai Jiao Tong University School of Medicine, Shanghai, China; 2 Institute of Human Development, Tzu Chi University, Hualien, Taiwan, China; 3 Yunnan Research Institute of Family Planning, Kunming, China; 4 MOE Key Laboratory of Contemporary Anthropology, Fudan University, Shanghai, China; 5 Department of Sociology and Anthropology, North Carolina State University, Raleigh, North Carolina, United States of America; 6 The People's Hospital of the Tibet Autonomous Region, Lhasa, Tibet Autonomous Region, China; 7 Health Training School of Cadres, Honghe Prefecture, Yunnan, China; 8 The People's Hospital of Xinjiang Uygur Autonomous Region, Wulumuqi, Xinjiang Uygur Autonomous Region, China; 9 Qinghai Hospital of Traditional Chinese Medicine, Xining, Qinghai, China; 10 Wulumuqi Epidemic Prevention Station, Wulumuqi, Xinjiang Uygur Autonomous Region, China; 11 Chinese National Human Genome Center at Shanghai, Shanghai, China; University of Illinois at Champaign-Urbana, United States of America

## Abstract

Completion of a survey of dermatoglyphic variables for all ethnic groups in an ethnically diverse country like China is a huge research project, and an achievement that anthropological and dermatoglyphic scholars in the country could once only dream of. However, through the endeavors of scientists in China over the last 30 years, the dream has become reality. This paper reports the results of a comprehensive analysis of dermatoglyphics from all ethnic groups in China. Using cluster analysis and principal component analysis of dermatoglyphics, it has been found that Chinese populations can be generally divided into a southern group and a northern group. Furthermore, there has been considerable debate about the origins of many Chinese populations and about proper assignment of these peoples to larger ethnic groups. In this paper, we suggest that dermatoglyphic data can inform these debates by helping to classify a Chinese population as a northern or southern group, using selected reference populations and quantitative methods. This study is the first to assemble and investigate dermatoglyphics from all 56 Chinese ethnic groups. It is fortunate that data on population dermatoglyphics, a field of physical anthropology, have now been collected for all 56 Chinese ethnic groups, because intermarriage between individuals from different Chinese ethnic groups occurs more frequently in recent times, making population dermatoglyphic research an ever more challenging field of inquiry.

## Introduction

Each person's set of fingerprints is different, but fingerprints for an individual remain stable over a lifetime. These characteristics have made fingerprints very useful as tools for law enforcement officials in many criminal cases. Fingerprints also vary considerably among different groups of people, and can be useful as tools for tracing individuals to particular populations. Because fingerprints are highly variable and genetically influenced, they have important significance for forensic science, anthropology, ethnology, genetics, and medicine [1], [3], [4].

Population dermatoglyphics is a field of research within physical anthropology. It focuses on the dermatoglyphics of different ethnic groups [1]–[4]. The investigation of population dermatoglyphics in China began in 1910 (Taiwan), and a total of more than fifty papers on dermatoglyphics were published prior to 1971, though they reported on only a limited number of dermatoglyphic variables [5]. Only a small number of research projects on dermatoglyphics were carried out in Mainland China before 1964, and large-scale investigation and research on dermatoglyphics did not begin until 1977 [6]–[10]. Over the past 30 years, through the endeavors of many dermatoglyphic researchers in China, we have jointly completed a grand research project on the dermatoglyphics of the Chinese people [6]–[27].

China has a population of 1.3 billion people, and a total of 56 different ethnic groups are recognized in the country [6], [10], [11], [16], [19]. The Han Chinese group has the greatest population with 1.2 billion members. We have now successfully completed an investigation and analysis of dermatoglyphics for all 56 Chinese ethnic groups. One result of this study has been a recognition that dermatoglyphics among Han Chinese show strong diversities.

Table 1 lists the geographic area, sample size and published references for all populations studied in China [6]–[30]. If a sample's abbreviation has an asterisk “*” after the name, it is a combined sample. In our study, an ethnic group may have samples from several populations, and the data from these populations are combined into one sample. The complete dataset of dermatoglyphic variables for the Chinese ethnic groups are listed in Table 2. This study is the first complete and comprehensive dermatoglyphic research for all 56 Chinese ethnic groups. It is fortunate that data on population dermatoglyphics have now been collected for all 56 Chinese ethnic groups, because intermarriage between individuals from different ethnic groups is more frequent in recent times, making population dermatoglyphic research an ever more challenging field of inquiry.

**Table 1:** Geographical area, sample size and references for samples and dermatoglyphic variables of Chinese samples and outgroups.

Ethnic group	Abbrev-iation	Province/Country	North latit-ude	East Long-tude	Males	Females	Total	TFRC	a-bRC	A	Lu	Lr	W	T/I	II	III	IV	H	Ref.
Achang	Achang-1	Yunnan	24.4	97.9	231	236	467	134.87	36.66	2.72	43.64	2.38	51.26	4.71	1.61	8.68	61.14	12.63	[11]
	Achang-2	Yunnan	24.8	98.2	287	290	577	133.07	38.73	3.31	52.37	2.79	41.53	5.37	1.21	17.42	77.30	15.17	[6]
	Achang-*						1044	133.88	37.80	3.05	48.46	2.61	45.88	5.07	1.39	13.51	70.07	14.03	
Bai	Bai----1	Yunnan	25.6	100.1	400	400	800	125.97	35.21	2.36	49.37	3.05	45.22	1.01	0.57	15.76	79.57	12.25	[11]
	Bai----2	Yunnan	26.0	99.9	500	500	1000	130.12	36.72	1.55	48.64	2.96	46.85	5.35	0.30	15.40	77.30	16.45	[6]
	Bai----*						1800	128.28	36.05	1.91	48.96	3.00	46.13	3.42	0.42	15.56	78.31	14.58	
Blang	Blang--1	Yunnan	22.0	100.8	187	204	391	132.68	34.96	2.29	51.84	2.72	43.15	2.67	1.02	14.39	82.68	14.64	[11]
	Blang--2	Yunnan	23.4	99.8	500	500	1000	125.55	33.81	1.72	51.33	1.52	45.43	2.75	0.95	9.20	71.00	12.70	[6]
	Blang--*						1391	127.55	34.13	1.88	51.47	1.86	44.79	2.73	0.97	10.66	74.28	13.25	
Bonan	Bonan--1	Gansu	35.7	102.8	126	41	167	137.96	39.21	1.06	47.89	2.89	48.16	4.73	0.00	6.57	51.81	20.50	[16]
	Bonan--2	Gansu	35.7	102.8	301	240	541	161.99	35.78	2.61	45.73	3.05	48.61	6.00	0.46	15.28	77.33	21.16	[16]
	Bonan--*						708	156.32	36.59	2.25	46.24	3.01	48.50	5.70	0.35	13.23	71.31	21.00	
Bouyei	Bouyei	Guizhou	26.6	106.7	230	218	448	132.99	36.68	0.85	44.80	2.25	52.10	3.24	0.89	12.83	65.86	8.93	[16]
Dai	Dai----1	Yunnan	24.4	97.9	300	300	600	130.00	38.25	2.38	47.92	2.27	47.43	1.25	0.75	13.83	72.58	10.67	[11]
	Dai----2	Yunnan	24.4	97.9	500	507	1007	125.37	37.50	4.00	53.68	3.18	39.14	2.78	1.54	14.35	67.87	9.63	[6]
	Dai----*						1607	127.10	37.78	3.39	51.53	2.84	42.24	2.21	1.25	14.16	69.63	10.02	
Daur	Daur	Xinjiang	46.7	82.9	500	500	1000	144.29	37.25	2.46	44.81	3.16	49.57	3.40	1.85	24.50	57.05	17.30	[16]
De'ang	De'ang-1	Yunnan	24.4	98.5	170	130	300	134.49	38.02	4.60	47.63	1.73	46.04	4.33	0.33	12.83	53.17	10.33	[11]
	De'ang-2	Yunnan	24.4	98.5	330	260	590	125.33	36.79	4.16	50.59	3.47	41.78	4.83	0.42	13.31	69.75	12.46	[6]
	De'ang-*						890	128.41	37.20	4.31	49.59	2.88	43.22	4.66	0.39	13.15	64.16	11.74	
Derung	Derung-1	Yunnan	27.7	98.6	100	98	198	124.35	34.60	4.14	44.19	6.26	45.41	4.55	0.50	12.88	78.28	8.59	[11]
	Derung-2	Yunnan	27.7	98.6	136	164	300	127.20	36.47	4.80	48.80	7.87	38.53	6.17	0.33	11.50	70.00	9.17	[6]
	Derung-*						498	126.07	35.73	4.54	46.97	7.23	41.26	5.53	0.40	12.05	73.29	8.94	
Dong	Dong---1	Guizhou	25.9	108.5	199	215	414	131.09	37.16	3.01	45.34	1.93	49.72	2.52	1.53	15.36	63.62	9.96	[16]
	Dong---2	Guangxi	25.8	110.1	340	330	670	140.18	36.90	2.31	49.18	2.84	45.67	3.97	1.72	13.41	69.36	15.43	[11]
	Dong---*						1084	136.71	37.00	2.58	47.71	2.49	47.22	3.42	1.65	14.15	67.17	13.34	
Dongxiang	DongX.	Gansu	35.6	103.3	307	75	382	142.88	38.02	2.29	48.50	3.18	46.03	8.81	1.74	11.75	55.03	18.58	[16]
Ewenki	Ewenki	Inner Mongolia	49.1	119.7	317	306	623	147.67	36.36	2.24	44.78	2.36	50.62	6.99	1.62	7.16	25.86	19.72	[16]
Gaoshan	GaoS.--1	Taiwan	23.6	121.6	50	50	100	162.21	40.20	1.20	38.50	2.60	57.70	8.00	0.00	14.50	79.00	14.50	[7]
	GaoS.--2	Taiwan	23.7	121.4	100	100	200	163.07	39.12	1.25	40.80	2.35	55.60	9.00	0.50	17.50	68.00	11.75	[8]
	GaoS.--*						300	162.78	39.48	1.24	40.03	2.43	56.30	8.67	0.33	16.50	71.67	12.67	
Gelao	Gelao	Guizhou	27.7	106.9	209	201	410	135.95	37.33	2.02	46.83	2.39	48.76	4.41	2.82	18.75	65.04	8.70	[16]
Gin	Gin----1	Guangxi	21.7	108.3	128	113	241	147.80	39.70	1.37	45.02	2.66	50.95	4.98	1.04	9.33	63.07	13.07	[11]
	Gin----2	Guangxi	21.7	108.3	270	230	500	140.81	38.95	1.78	45.24	2.78	50.20	3.10	0.60	9.10	61.30	7.00	[16]
	Gin----*						741	143.08	39.19	1.65	45.17	2.74	50.44	3.71	0.74	9.17	61.88	8.97	
Han	Han----1	Taiwan	25.0	121.5	100	100	200	151.26	39.38	2.15	43.95	2.40	51.50	5.75	3.00	20.50	70.50	19.50	[9]
	Han----2	Taiwan	25.0	121.5	100	100	200	143.38	40.01	2.25	47.85	2.30	47.60	9.00	1.25	20.50	75.75	20.50	[10]
	Han----3	Shanxi	33.6	109.1	134	133	267	102.40	32.34	3.71	43.61	2.32	50.36	11.28	1.13	6.02	62.97	13.16	[16]
	Han----4	Anhui	31.3	118.4	220	162	382	136.29	37.23	2.88	45.11	2.17	49.84	4.84	2.62	14.14	65.32	14.13	[12]
	Han----5	Guizhou	27.7	106.9	204	209	413	135.89	39.70	2.20	44.31	1.77	51.72	7.53	1.88	14.59	68.35	5.76	[11]
	Han----6	Liaoning	41.1	121.1	250	250	500	126.34	33.60	3.64	48.32	2.62	45.42	5.50	2.20	5.20	65.50	10.10	[16]
	Han----7	Shanghai	31.2	121.4	309	284	593	133.25	38.09	2.60	45.41	2.39	49.60	11.41	0.42	11.96	58.32	18.79	[13]
	Han----8	Sichuan	28.8	105.4	367	327	694	150.97	38.96	2.33	44.99	2.58	50.10	8.27	0.92	11.58	56.18	11.44	[11]
	Han----9	Inner Mongolia	49.0	119.0	456	456	912	127.86	31.35	2.10	47.67	2.64	47.59	4.35	3.26	21.14	77.94	11.89	[14]
	Han---10	Shanghai	31.2	121.4	520	520	1040	143.63	38.05	2.05	44.65	2.44	50.86	8.67	0.87	14.66	73.46	17.26	[6]
	Han---11	Jiangsu	34.2	117.1	582	508	1090	129.87	34.07	2.06	47.14	2.06	48.74	5.00	1.69	15.92	65.00	11.65	[16]
	Han---12	Jiangsu	32.0	118.7	698	483	1181	128.22	38.53	2.21	45.11	3.20	49.48	8.89	1.61	15.63	66.70	12.31	[16]
	Han---13	Shanghai	31.2	121.4	640	560	1200	131.10	36.90	0.90	43.90	2.70	52.50	3.00	2.15	14.40	68.35	11.30	[15]
	Han---14	Tianjin	39.1	117.2	642	638	1280	141.92	40.04	1.88	46.87	2.44	48.81	10.62	2.07	18.44	74.05	17.69	[17]
	Han---15	Shanghai	31.2	121.4	640	661	1301	126.77	35.77	3.45	43.65	2.54	50.36	10.14	1.69	14.72	65.95	16.33	[18]
	Han----*						11253	133.68	36.83	2.29	45.49	2.51	49.71	7.60	1.77	15.05	68.01	13.94	
Hani	Hani---1	Yunnan	22.0	100.7	210	210	420	118.32	36.03	3.19	51.83	2.84	42.14	3.93	0.72	15.72	72.27	12.14	[11]
	Hani---2	Yunnan	23.1	102.7	520	167	687	135.90	35.99	1.41	49.87	2.88	45.84	0.80	0.80	15.65	82.68	11.21	[19]
	Hani---3	Yunnan	23.4	102.8	500	500	1000	137.57	38.49	2.54	51.88	2.57	43.01	6.90	0.70	14.35	79.05	20.65	[6]
	Hani---*						2107	133.19	37.18	2.30	51.21	2.73	43.76	4.32	0.74	15.05	78.88	15.88	
Hezhen	Hezhen	Heilongjiang	46.8	134.0	86	80	166	142.14	35.35	3.19	47.95	2.05	46.81	12.35	1.81	21.99	51.20	11.14	[16]
Hui	Hui----1	Hainan	17.8	109.2	183	38	221	145.47	38.38	1.85	54.51	2.34	41.30	6.13	0.00	6.12	49.00	9.08	[16]
	Hui----2	Anhui	33.8	115.7	200	200	400	138.79	37.12	2.60	49.87	2.38	45.15	7.75	1.00	19.25	69.75	15.00	[16]
	Hui----3	Yunnan	24.1	102.7	200	200	400	130.03	36.28	3.10	47.20	1.85	47.85	4.13	0.63	11.25	53.88	10.63	[11]
	Hui----4	Gansu	35.6	103.1	364	170	534	157.09	38.98	1.64	44.66	2.70	51.00	6.94	0.47	8.67	47.56	20.53	[16]
	Hui----5	Inner Mongolia	40.8	111.7	309	411	720	128.10	36.00	2.75	49.34	2.38	45.53	4.97	1.84	16.48	62.57	15.98	[14]
	Hui----6	Ningxia	38.4	106.2	431	500	931	127.25	36.79	4.40	48.50	2.20	44.90	5.90	0.10	26.20	80.80	19.70	[16]
	Hui----7	Yunnan	25.5	103.2	500	500	1000	129.21	37.39	2.19	51.92	2.80	43.09	2.65	0.30	8.00	76.75	16.25	[6]
	Hui----*						4206	133.97	37.14	2.81	49.29	2.43	45.47	5.12	0.62	14.85	67.21	16.48	
Jingpo	Jingpo-1	Yunnan	24.4	97.9	254	242	496	135.08	38.09	2.56	47.16	2.05	48.23	1.31	0.71	15.43	72.38	7.46	[11]
	Jingpo-2	Yunnan	24.5	98.5	500	500	1000	131.45	35.80	2.44	51.52	3.41	42.63	3.30	1.30	11.70	67.60	10.90	[6]
	Jingpo-*						1496	132.65	36.56	2.48	50.07	2.96	44.49	2.64	1.10	12.94	69.18	9.76	
Jino	Jino---1	Yunnan	22.0	100.8	120	120	240	122.01	35.74	2.96	54.04	2.50	40.50	3.13	0.83	10.00	80.00	17.50	[11]
	Jino---2	Yunnan	22.0	100.8	395	439	834	123.82	36.42	3.43	55.75	2.22	38.60	1.74	0.42	6.54	78.06	15.05	[6]
	Jino---*						1074	123.42	36.27	3.33	55.37	2.28	39.02	2.05	0.51	7.31	78.49	15.60	
Kazak	Kazak	Xinjiang	43.8	87.6	500	500	1000	134.11	37.86	2.61	52.52	4.23	40.64	9.20	2.55	30.40	61.75	34.70	[6]
Kirgiz	Kirgiz	Xinjiang	39.5	76.0	500	500	1000	139.47	38.88	2.81	49.10	3.77	44.32	9.55	1.95	25.25	63.35	31.70	[6]
Korean	Korean-1	Jilin	42.9	129.5	200	200	400	142.75	36.10	3.00	49.43	8.17	39.40	7.75	1.85	6.70	41.65	16.70	[20]
	Korean-2	Jilin	42.9	129.5	205	277	482	136.13	36.00	1.21	48.90	2.40	47.49	7.67	1.77	6.49	41.73	16.94	[16]
	Korean-3	Inner Mongolia	46.0	122.0	270	267	537	136.74	37.42	2.32	51.66	2.82	43.20	4.57	0.84	11.82	73.65	17.97	[14]
	Korean-4	Liaoning	41.6	123.4	300	300	600	102.22	30.62	3.08	51.50	2.48	42.94	2.00	0.83	13.58	56.33	8.25	[16]
	Korean-*						2019	127.53	34.80	2.42	50.51	3.68	43.39	5.18	1.26	10.06	54.54	14.58	
Lahu	Lahu---1	Yunnan	21.9	101.4	91	87	178	141.34	35.04	1.12	57.47	2.75	38.66	5.90	1.12	8.71	80.34	19.66	[11]
	Lahu---2	Yunnan	22.5	99.9	90	110	200	153.32	36.08	2.10	34.55	1.20	62.15	3.00	0.25	15.00	61.25	8.00	[21]
	Lahu---3	Yunnan	21.9	101.4	268	300	568	148.61	34.92	0.95	41.90	1.69	55.46	4.94	1.06	18.35	70.90	7.14	[16]
	Lahu---4	Yunnan	22.5	99.9	480	500	980	143.16	35.85	1.26	45.67	1.94	51.13	2.70	0.82	29.49	64.49	4.85	[19]
	Lahu---*						1926	145.65	35.52	1.24	44.49	1.87	52.40	3.69	0.86	22.78	67.51	7.22	
Lhoba	Lhoba	Tibet	29.2	94.1	142	190	332	147.05	38.40	1.47	41.72	1.54	55.27	8.58	0.15	12.95	82.53	14.31	[6]
Li	Li-----1	Hainan	18.6	109.7	258	270	528	133.76	36.48	2.66	48.84	2.20	46.30	2.48	0.79	5.24	42.63	12.81	[16]
	Li-----2	Hainan	19.9	109.6	406	152	558	142.88	37.08	2.87	46.04	2.90	48.19	5.29	2.96	19.00	72.67	16.22	[16]
	Li-----*						1086	138.45	36.79	2.77	47.40	2.56	47.27	3.92	1.90	12.31	58.06	14.56	
Lisu	Lisu---1	Yunnan	24.3	97.9	110	95	205	144.41	38.26	1.56	41.90	1.22	55.32	1.46	0.00	6.10	60.98	1.95	[11]
	Lisu---2	Yunnan	25.9	98.7	500	283	783	137.56	38.33	1.98	49.95	3.83	44.24	2.17	0.57	10.92	73.95	7.92	[6]
	Lisu---*						988	138.98	38.32	1.89	48.28	3.29	46.54	2.02	0.45	9.92	71.26	6.68	
Man	Man	Liaoning	40.6	120.6	242	230	472	126.03	33.18	2.01	49.06	2.78	46.15	6.78	0.85	8.37	51.80	16.63	[16]
Maonan	Maonan	Guangxi	24.8	108.2	240	240	480	130.63	36.31	3.46	52.83	2.42	41.29	3.75	2.71	13.75	67.92	14.90	[11]
Miao	Miao---1	Hainan	18.6	109.7	181	150	331	140.12	37.15	1.99	53.44	1.81	42.76	4.25	1.21	11.65	57.74	10.33	[16]
	Miao---2	Sichuan	28.1	105.7	188	167	355	131.86	38.69	4.00	60.88	2.90	32.22	1.42	1.96	13.92	59.81	11.65	[11]
	Miao---3	Guizhou	26.6	108.0	221	182	403	133.05	38.94	1.49	44.89	2.16	51.46	3.44	1.49	11.43	74.08	8.35	[16]
	Miao---*						1089	134.81	38.31	2.46	52.70	2.30	42.54	3.03	1.56	12.31	64.46	10.03	
Monba	Monba	Tibet	27.9	91.9	101	116	217	157.91	39.46	1.07	39.20	1.80	57.93	7.14	0.00	17.05	72.81	25.58	[6]
Mongol	Mongol-1	Inner Mongolia	42.2	118.9	300	300	600	123.70	32.37	2.53	46.30	2.47	48.70	2.33	1.42	15.67	58.92	14.25	[16]
	Mongol-2	Yunnan	24.0	102.7	313	413	726	133.40	40.05	2.39	55.89	1.83	39.89	5.51	0.69	14.12	71.07	7.02	[19]
	Mongol-3	Inner Mongolia	46.0	122.0	515	553	1068	143.34	35.97	1.84	45.53	2.83	49.80	7.51	2.33	24.47	67.01	15.02	[14]
	Mongol-*						2394	135.40	36.31	2.18	48.86	2.44	46.52	5.61	1.60	19.13	66.21	12.40	
Mulam	Mulam--1	Guangxi	24.7	108.9	226	261	487	126.41	36.99	4.33	48.52	2.57	44.58	7.91	1.54	16.22	85.12	14.68	[16]
	Mulam--2	Guangxi	24.7	108.9	260	260	520	135.25	36.93	2.67	51.06	1.87	44.40	6.73	1.45	15.96	72.50	13.56	[11]
	Mulam--*						1007	130.97	36.96	3.47	49.83	2.21	44.49	7.30	1.49	16.09	78.60	14.10	
Naxi	Naxi---1	Yunnan	26.8	100.2	310	310	620	132.02	36.99	1.89	46.52	2.16	49.43	2.26	0.97	16.05	81.54	13.55	[16]
	Naxi---2	Yunnan	26.8	100.2	408	420	828	132.21	37.77	1.10	43.40	2.14	53.36	5.26	0.91	19.44	70.53	12.34	[19]
	Naxi---*						1448	132.13	37.44	1.44	44.73	2.15	51.68	3.98	0.94	17.99	75.24	12.86	
Nu	Nu-----1	Yunnan	26.4	99.2	73	65	138	132.50	36.93	1.74	45.79	1.82	50.65	6.16	0.36	9.05	91.31	10.14	[11]
	Nu-----2	Yunnan	25.9	98.7	175	176	351	149.03	39.08	1.34	45.89	2.71	50.06	6.41	0.43	16.81	73.79	8.40	[6]
	Nu-----*						489	144.37	38.47	1.45	45.86	2.46	50.23	6.34	0.41	14.62	78.73	8.89	
Oroqen	Oroqen	Inner Mongolia	51.7	126.6	184	238	422	146.34	35.83	2.41	45.86	2.19	49.54	10.65	1.01	10.91	25.20	18.36	[16]
Primi	Primi	Yunnan	26.4	99.2	159	138	297	157.84	39.27	1.65	38.08	1.42	58.85	12.96	1.35	14.14	86.53	8.59	[11]
Qiang	Qiang--1	Sichuan	31.6	103.8	262	149	411	145.97	39.29	1.66	43.78	2.80	51.76	7.79	0.89	7.54	64.55	9.94	[16]
	Qiang--2	Sichuan	31.6	103.8	296	272	568	164.32	40.14	2.10	48.34	2.68	46.88	10.77	1.49	18.74	63.57	11.56	[16]
	Qiang--*						979	156.62	39.78	1.91	46.43	2.73	48.93	9.52	1.24	14.04	63.98	10.88	
Russ	Russ	Xinjiang	43.8	87.6	31	25	56	143.87	38.45	3.93	56.97	3.39	35.71	7.14	1.79	25.89	54.46	15.18	[22]
Salar	Salar	Qinghai	35.8	102.4	102	102	204	149.40	40.21	1.72	44.85	4.95	48.48	8.58	1.72	19.36	75.98	25.49	[23]
She	She	Zhejiang	28.5	119.9	270	155	425	134.20	37.21	3.70	49.36	2.68	44.26	11.31	1.50	15.20	70.70	13.20	[16]
Sui	Sui----1	Guizhou	26.0	107.8	135	170	305	145.40	36.32	1.79	43.32	2.05	52.84	7.33	2.61	16.13	77.03	16.45	[16]
	Sui----2	Guizhou	26.0	107.8	206	207	413	136.60	37.07	1.77	41.55	1.91	54.77	2.54	1.57	11.02	72.28	13.44	[16]
	Sui----*						718	140.34	36.75	1.78	42.30	1.97	53.95	4.57	2.01	13.19	74.30	14.72	
Tajik	Tajik	Xinjiang	37.7	75.2	562	500	1062	134.26	39.00	6.57	47.49	2.65	43.29	4.24	3.30	28.25	50.75	26.93	[16]
Tatar	Tatar	Xinjiang	43.8	87.6	29	24	53	146.58	41.35	2.64	59.62	4.91	32.83	4.72	2.83	39.62	59.43	41.51	[24]
Tibetan	T.B.---1	India	28.0	77.0	156	150	306	148.10	39.82	1.48	41.98	2.08	54.46	4.18	0.49	9.11	63.92	18.59	[16]
	T.B.---2	Tibet	29.6	91.1	182	189	371	145.95	39.30	1.20	38.13	1.45	59.22	4.75	0.55	4.07	50.81	16.96	[16]
	T.B.---3	Sichuan	33.0	101.7	223	181	404	148.03	39.72	1.88	41.68	2.00	54.44	7.35	0.00	18.25	75.18	14.70	[16]
	T.B.---4	Sichuan	32.4	104.4	246	242	488	153.56	37.12	1.97	42.25	2.52	53.26	13.42	0.71	10.66	66.19	18.65	[16]
	T.B.---5	Tibet	29.6	91.1	226	291	517	142.31	39.11	1.97	41.45	1.72	54.86	6.93	1.07	9.49	72.60	17.91	[25]
	T.B.---6	Sichuan	31.8	102.4	341	326	667	161.49	39.79	1.87	47.45	3.60	47.08	9.98	0.70	14.26	72.40	10.06	[11]
	T.B.---7	Gansu	34.9	102.9	500	500	1000	168.10	34.95	3.04	44.71	3.00	49.25	11.20	1.60	6.00	63.95	12.30	[16]
	T.B.---8	Tibet	29.6	91.1	500	500	1000	143.62	38.01	1.18	41.74	2.73	54.35	6.10	0.60	11.70	82.00	25.90	[6]
	T.B.---*						4753	153.00	38.01	1.92	42.92	2.57	52.59	8.44	0.82	10.31	70.03	17.08	
Tu	Tu	Qinghai	36.8	101.9	106	108	214	143.47	39.66	1.92	50.98	2.90	44.20	7.95	1.64	19.16	73.36	21.96	[26]
Tujia	Tujia	Sichuan	28.4	108.9	265	240	505	120.04	38.54	2.43	45.84	1.86	49.87	8.51	1.48	12.97	60.79	16.43	[11]
Uygur	Uygur	Xinjiang	43.8	87.6	500	500	1000	138.09	37.27	2.51	50.28	3.75	43.46	14.90	4.70	39.15	62.00	33.10	[6]
Uzbek	Uzbek	Xinjiang	46.8	82.8	600	600	1200	152.00	38.00	3.46	49.39	2.76	44.39	5.91	5.63	45.67	54.38	27.00	[16]
Va	Va-----1	Yunnan	22.7	99.4	416	354	770	137.78	37.63	2.01	56.36	2.09	39.54	2.80	0.58	16.28	77.56	9.75	[19]
	Va-----2	Yunnan	23.1	99.2	500	400	900	139.60	38.20	2.34	57.61	2.82	37.23	2.67	1.06	14.39	73.67	13.67	[16]
	Va----*						1670	138.76	37.94	2.19	57.03	2.48	38.30	2.73	0.84	15.26	75.46	11.86	
Xibe	Xibe	Xinjiang	43.7	81.5	500	500	1000	146.50	39.00	1.81	45.39	2.63	50.17	7.50	1.80	21.05	64.95	21.00	[16]
Yao	Yao----1	Guangxi	24.9	107.7	350	140	490	123.14	35.69	2.51	51.63	1.96	43.90	12.45	0.92	20.71	52.25	7.55	[27]
	Yao----2	Guangxi	24.1	107.2	376	168	544	128.45	34.00	3.20	43.58	2.41	50.81	1.47	2.48	13.79	65.07	7.54	[16]
	Yao----*						1034	125.93	34.80	2.87	47.39	2.20	47.54	6.67	1.74	17.07	58.99	7.54	
Yi	Yi-----1	Sichuan	28.0	102.8	180	160	340	150.63	40.38	2.12	52.50	2.76	42.62	6.18	1.18	14.27	79.27	16.91	[11]
	Yi-----2	Yunnan	25.0	102.7	200	200	400	139.15	39.42	1.33	52.50	2.47	43.70	2.25	0.25	16.13	79.00	13.38	[11]
	Yi-----3	Yunnan	25.0	101.5	250	250	500	135.08	37.80	1.10	43.60	1.52	53.78	4.00	0.00	12.80	67.20	17.80	[16]
	Yi-----4	Sichuan	27.7	102.8	434	71	505	153.48	41.34	2.00	46.37	3.09	48.54	5.73	1.16	7.73	48.79	11.50	[16]
	Yi-----5	Yunnan	24.7	103.2	500	500	1000	135.38	38.90	1.62	51.20	2.82	44.36	2.00	0.20	16.15	66.60	9.50	[6]
	Yi-----*						2745	141.09	39.41	1.62	49.28	2.57	46.53	3.60	0.47	13.75	66.81	12.86	
Yugur	Yugur	Gansu	38.8	99.6	185	151	336	147.40	40.71	2.03	44.30	2.29	51.38	9.05	1.63	18.99	55.79	25.07	[16]
Zhuang	Zhuang-1	Guangxi	23.8	106.6	298	202	500	133.40	37.79	3.98	48.20	2.00	45.82	5.50	2.60	25.00	75.60	14.30	[16]
	Zhuang-2	Guangxi	23.1	107.1	287	283	570	129.55	36.27	2.75	52.09	2.63	42.53	5.30	1.70	15.10	68.50	18.50	[11]
	Zhuang-*						1070	131.35	36.98	3.32	50.27	2.34	44.07	5.39	2.12	19.73	71.82	16.54	
Mang	Mang	Yunnan	22.7	103.2	124	110	234	118.42	36.71	4.10	62.44	2.35	31.11	6.41	0.00	8.98	64.10	6.62	[16]
Gin	Gin-VieT	Vietnam	21.0	106.0	66	69	135	128.00	36.30	5.40	46.90	1.70	46.00	0.40	1.10	12.30	65.20	9.30	[30]
Africans	Africans	South Africa			200	200	400	124.72	37.62	4.85	64.70	2.70	27.75	1.13	9.40	41.75	83.50	34.13	[28], [29]
Caucasians	Caucasia	USA			200	200	400	131.65	41.35	7.95	61.45	4.40	26.20	7.10	2.50	37.65	45.85	35.20	[3]

* A “*” indicates a combined sample for an ethnic group. Among the 56 ethnic groups, 31 are represented by more than one sample and 25 by only one sample. English names of these ethnic groups were based on Chinese Encyclopedia - Ethnic Groups (Encyclopedia Publisher, Beijing, Shanghai, Jun. 1986), and these English names were arranged in alphabetical order. The sample sizes for Bouyei, Dong-1, Dong-2, Ewenki, Han-5, Miao-2, Miao-3, Oroqen and Yugur are those from fingerprints since sample sizes for each dermatoglyphic variable may not be the same in a population. There are 1509 fingers in 151 Yugur females with one of them having an injured middle finger of the right hand. The following data are published for the first time: II of GaoS.-1, II and H of GaoS.-2, III of Han-1, II and III of Han-2, IV and H of Salar, T/I of Tatar, and II and III of Tu. Data II of Han-13 was kindly provided by Dr. Hui Li. The frequency of II of Russ from females was originally reported incorrectly as 20%. A total of 34 samples were investigated by authors of this paper, and these samples are Achang-2, Bai-2, Blang-2, Dai-2, De'ang-2, Derung-2, GaoS.-1-2, Han-1-2-10-15, Hani-2-3, Hui-7, Jingpo-2, Jino-2, Kazak, Kirgiz, Lahu-4, Lhoba, Lisu-2, Monba, Mongol-2, Naxi-2, Nu-2, Russ, Salar, Tatar, T.B.-8, Tu, Uygur, Va-1, and Yi-5. Among the 56 ethnic groups, we studied 29 (51.79%). Among the 121 populations, we studied 34 (28.10%).

**Table 2:** Principal Component analysis of 29 PM & 2 SM and Han-10 of Shanghai.

No.	PM^a^ & SM^b^	ethnic groups	PCI z_i1_	PCII z_i2_	PCIII z_i3_	PCIV z_i4_
1	PM-S^c^	Achang-2	0.2399	−0.1747	0.6042	−0.4677
2	PM-S	Bai----2	−0.0529	−0.4894	0.5706	−0.2354
3	PM-S	Blang--2	−0.1020	−1.3572	−0.4173	1.4570
4	PM-N^d^	Bonan--2	−0.2290	0.2155	0.5376	−0.1416
5	PM-S	Dai----2	0.4040	−0.7465	−0.1697	−0.9512
6	PM-N	Daur	0.0015	0.2013	0.1736	−0.1806
7	PM-S	De'ang-2	0.2649	−0.6173	−0.2867	−1.2678
8	PM-S	Dong---2	0.0086	−0.3368	0.2246	−0.0644
9	PM-N	Dongxiang	−0.0867	0.3428	−0.5854	−0.5603
10	PM-N	Ewenki	−0.3760	0.2720	−1.9678	0.1914
11	PM-S	Hani---3	0.0682	0.0177	0.6914	−0.1180
12	PM-N	Hezhen	−0.1786	0.2268	−1.7250	0.9234
13	PM-N	Hui----4	−0.4375	0.5904	−0.2046	−0.6614
14	PM-S	Jingpo-2	0.1461	−0.8400	−0.2555	−0.7281
15	PM-S	Jino---2	0.2725	−1.1702	0.1397	−0.0252
16	PM-N	Korean-2	−0.2645	−0.2273	−1.5184	0.4181
17	PM-N	Lhoba	−0.6095	0.0769	1.1188	0.7162
18	PM-S	Lisu---2	0.0533	−0.6645	0.7987	−1.8339
19	PM-S	Maonan	0.2757	−0.5810	−0.3408	0.5417
20	PM-N	Monba	−0.6430	0.8287	1.5115	0.6402
21	PM-N	Mongol-3	−0.1168	0.1244	−0.0349	0.6394
22	PM-S	Mulam--2	−0.0156	−0.3476	−0.0309	0.7808
23	PM-N	Oroqen	−0.4078	0.4133	−2.4687	0.3475
24	PM-N	Qiang--2	−0.2862	0.8945	0.2502	−0.5886
25	PM-N	Titeban-8	−0.3730	0.2263	1.4243	0.1645
26	PM-N	Xibe	−0.1612	0.6358	0.5821	0.2102
27	PM-S	Yi-----5	0.0145	−0.5395	0.7167	−0.9459
28	PM-N	Yugur	−0.2458	1.1025	0.4154	0.1799
29	PM-S	Zhuang-2	0.1841	−0.4111	−0.2357	0.3549
30	SM	Africans	1.5700	0.4936	0.8020	2.9551
31	SM	Caucasians	1.3888	1.6194	−0.8029	−1.8662
32	**?** e	Han----10	−0.3063	0.2212	0.4827	0.1162

a PM - population marker.

b SM - supervisory marker.

c N - northern population.

d S - southern population.

e ? - simulated population remaining to be determined.

## Results

### Results from the Cluster Analysis of 56 Chinese Ethnic Groups

**Figure 1:**
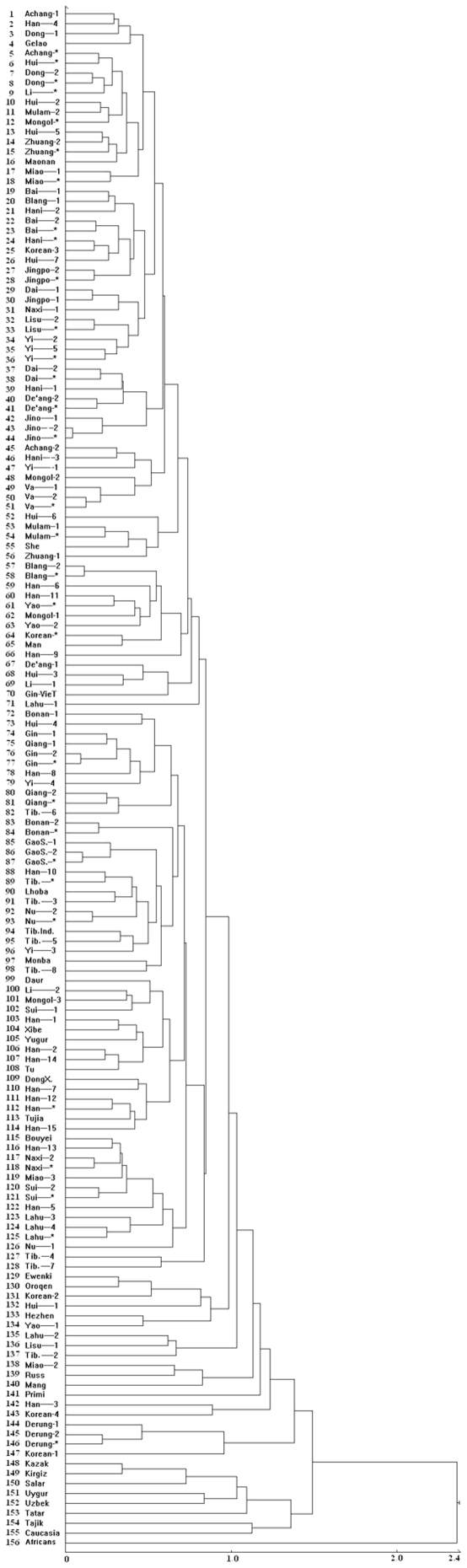
Cluster tree for 156 samples (including 56 Chinese ethnic groups, Africans, Caucasians and Gin Vietnamese). There are 156 populations numbered from top to bottom 1–156. In the figure, there is a southern group (SG) (1–71) and a northern group (NG) (72–154). There are two outgroups: Caucasians (155) and Africans (156). Gin-Vietnamese (70) clusters with the SG. The cluster tree was drawn using the average linkage method.

Figure 1 shows the results of a cluster analysis performed on the 156 samples. These samples include 122 population samples, 31 combined samples, and Africans, Caucasian Americans and Gin Vietnamese. The cluster analysis shows two major sub-clusters: a southern group (1–71) and a northern group (72–154), demonstrating that all ethnic groups in China do not share similar physical characters.

#### Southern group (1–71)

This group contains 70 Chinese samples (excluding Gin-Vietnamese), and includes only nine samples from northern China. Fifty six percent of these northern samples (5 samples) are concentrated in clusters 57–66. Geographically, this can be treated as an area of transition between the southern group and the northern group, or as a mixed area. For the physical characters of dermatoglyphics, there is a process of gradual diffusion from south to north or from north to south. Migration and mixing of many ethnic groups are still restricted by geographical barriers.

#### Northern group (72–154)

This group contains 83 samples. Clusters 115–126 contain samples from southern China. Therefore, this cluster could be seen as a transition area between the northern group and the southern group. In the northern group, there are several ethnic groups from Xinjiang province (Kazak, Kirgiz, Uygur, Uzbek, Tatar, Tajik) and Salar of Qinghai Province. These seven samples constitute a cluster by themselves. With the exception of the Salar, the fingerprint frequency of whorl (W) among these six Xinjiang samples is significantly lower than the frequency of loop (L) (*p*<0.01), and the frequency of true pattern in the third interdigital area (III) in the hands is higher than 20%. Our Xingjiang samples express clear characters similar to the peoples of Central and Western Asia, and they could be treated singly as a “northwest group”.

#### Some Experience

Africans and Caucasian Americans, working as outgroups, express clear and suitable positions on the cluster tree. Gin-Vietnamese cluster in the southern Chinese group. Caucasian Americans first cluster with the Tajik and then cluster with northwest samples. Africans form the most peripheral cluster.

Combined samples representing 31 ethnic groups are included in the cluster analysis. The frequencies of their dermatoglyphic variables were calculated using the population size of each population sample. The combined samples tend to show a general picture for a specific ethnic group.

Sichuan is a province with many minorities of large population size in southwestern China. Ten (Han-8, Miao-2, Qiang-1-2-*, Tibetan-6-3-4, Tujia, Yi-4) of 11 samples from this province cluster in the northern group (including the Qiang combined sample) with only one sample (Yi-1) clustering in the southern group. During the past three centuries, the population in Sichuan has increased from 100 thousand to 100 million. Most likely, Sichuan is a place of migration and fusion of peoples.

Han Chinese are represented by 16 samples (including combined samples): 4 samples (Han-4-6-11-9) cluster in the southern group and 12 samples (Han-8-10-1-2-14-7-12-*-15-13-5-3) cluster in the northern group. Han-2 and Han-14 are neighboring samples in the northern group on the cluster tree, but Han-2 and Han-14 were collected separately from the south and north. Two samples (Han-6-9) were collected from the north but cluster in the southern group. Nine samples (Han-8-10-1-2-7-12-15-13-5) that were collected from the south actually cluster in the northern group. Three samples of Han Chinese in Shanghai (Han-10-15-13), with each sample having more than 1000 persons, all cluster in the northern group. Within clusters 109–114, there is a section containing many samples of Han Chinese. Samples of Han Chinese do not cluster into a single group. Han Chinese is the ethnic group with the largest population in China and throughout the world. Cluster analysis indicates that Han Chinese samples from different places (east, northwest, northeast and southwest) tend to cluster together as a group with local minorities. Therefore, the dermatoglyphic characters of Han Chinese express strong nationwide diversities.

Many large migrations through history, including migrations from south to north and from north to south, as well as migrations relating to the opening of the Silk Road for interchange between the east and the west, have divided the original ethnic groups into different populations. For example, migratory populations such as Mongol-2 and Hui-2-7-3 who migrated from northern China to southern China cluster with a neighboring ethnic group (southern group). This indicates correlations between physical characters of dermatoglyphics and geographical areas. Clearly, there can be large differences between migratory populations and the original population within the same ethnic group.

All nine Tibetan samples (including combined samples) cluster with the northern group although they are geographically located in southwestern China. There are five Tibetan samples (Tibetan-*-3-Ind.-5-8) in cluster 85-98 where Tibetan populations are relatively concentrated. Tibetan dermatoglyphics shows characters of the northern group. Therefore, it seems that Tibetans are a northern group and not a “southern group from India” as has been suggested by scholars. It seems likely that Tibetans originated from the ancient Qiang people in northern China.

Tibetan-4 is a population whose origin is up for debate, and they are known as Baima Tibetan people in Sichuan province. On the cluster tree, Tibetan-4 clusters with Gansu Tibetan (T.B.-7) in northwestern China. This suggests that there is a difference between Baima Tibetan People and Tibetan people living in Tibet. Tibetan migrants in India (T.B.-1) cluster with the Tibetan sample from the Lhasa area (T.B.-5), expressing a close relationship between these two populations.

Mang is a population that has not yet been assigned to an ethnic group. In the cluster tree, Mang clusters with Miao-2 and Russ (138,139). This result does not help to assign them to a particular ethnic group.

Regarding the Miao samples, Miao-1 was collected from Hainan Island (province) and clusters in the southern group, and Miao-3-2 in Sichuan and Guizhou provinces cluster in the northern group. This result may be explained by evolution of physical characters occurring in populations that are isolated in an island setting.

Minnan Han Chinese (Han-2) is the largest population in Taiwan. Their dermatoglyphics are similar to the mainland northern group [10]. Minnan people in Taiwan come from the southern part of Fujian Province, and Minnan people in Fujian originate from northern China. The dermatoglyphics of Hakka Han Chinese in Taiwan (Han-1) are also similar to the northern group [9].

Taiwan aborigines (Gaoshan ethnic group) are represented by two samples in this research: Amis (GaoS.-2) [8] with a large population (167 thousand) and the Kavalan sample (GaoS.-1) [7] with a very small population (about 800). Taiwanese aboriginal samples (GaoS.-1-2-*) all cluster in the northern group. It is not clear why they do not share a close relationship with southern Chinese minorities.

Yi people in Yunnan Province are represented by two samples in the analysis: Samei (Yi-2) and Luoluobo (Yi-3). They separately cluster in the southern group and the northern group. Differences between these Yi populations are obvious.

Six samples, including Bai-2, Yi-5, Jino-2, Hani-2-3, and Blang-2 studied by Haiguo Zhang and colleagues, and five different samples collected from the same ethnic groups (Bai-1,Yi-2, Jino-1, Hani-1, Blang-1) studied by Anlu Jin and colleagues, all cluster in the southern group. Also, Derung-2 [6] studied by Haiguo Zhang and colleagues and Derung-1 [11] studied by Anlu Jin and colleagues both cluster in the northern group. Scholars from different research teams can obtain similar results using different samples collected from the same ethnic groups in Yunnan province. This fact demonstrates that the technical analysis [1]–[3] standard and variables standard [6], [19], [31] required by the Chinese Dermatoglyphics Association (CDA) has great value and effectiveness.

## Discussion

Dermatoglyphic characteristics can divide Chinese populations into a southern group and a northern group, taking the Yangtze River or 30^0^–33^0^ latitude as the boundary. This conjecture is similar to the results of dermatoglyphic research conducted in 1998 [6]. Previous studies from anthropometrics, HLA and immunoglobulin have also suggested that Chinese ethnic groups can be divided into northern and southern groups, and that they may be of different origins. [16]. Since there are great differences between the southern and northern groups, it is better to use data collected from local ethnic groups as references for medical applications and genetic studies.

There has been much debate about the origins of many Chinese populations and about proper assignment of these peoples to ethnic groups. Dermatoglyphic data can inform these debates by helping to classify a population as a northern or southern group. In order to make such assignments, we selected 29 samples from the dataset as reference populations (as population marker, PM). The 29 reference populations were limited to northern ethnic groups that actually cluster into the northern group, and southern ethnic groups that actually cluster into the southern group. In addition, preference was given to populations with larger sample sizes. Two outgroups, Africans and Caucasian Americans (as supervisory marker, SM), were also used to make such assignments.

**Figure 2:**
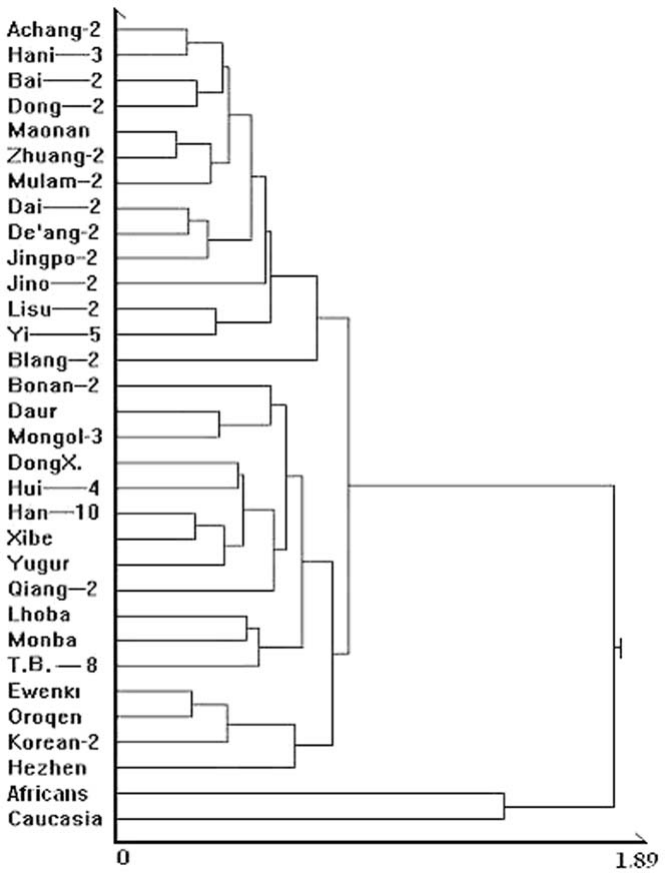
Cluster tree for Shanghai Han (Han-10), 29 reference populations (PM) and 2 outgroups (SM). Shanghai Han clustered with the northern group. This figure was drawn based on cluster analysis using the average linkage method.

There are 11 clustering methods available for cluster analysis in SAS software. If a clustering method is suitable for assigning a population to the northern or southern group, it should output 29 reference populations and 2 outgroups divided into four groups in the cluster tree: a southern group, a northern group, an African group and a Caucasian group. After selection, we found five usable clustering methods: Average linkage, complete or longest distance method, flexible-beta method, McQuitty's similarity method, and Ward's minimum-variance method. All these methods can classify 31 samples into 4 large groups. Although each of these five methods results in a different position (Y axis) in the clustering figure or a different clustering distance (X axis) for each population, the positions of the populations within the four groups is relatively stable. Figure 2 is an example of the results for the average linkage method, from which the cluster figure for 31 samples and the Han Chinese in Shanghai (Han-10) has been drawn. The results from the cluster analysis show that the Han-10 sample should be assigned to the northern group.

**Figure 3:**
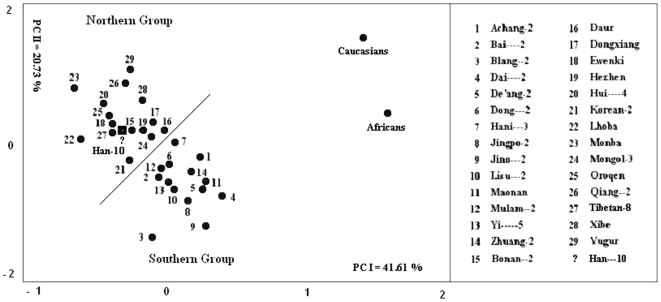
Scatter gram for principal component analysis of Shanghai Han (Han-10), 29 reference populations (PM) and 2 outgroups (SM). This figure was drawn based on standardized principal component scores. Shanghai Han (indicated by the “?”) stays in the northern group.

We also conducted principal component analysis on these 32 samples, and used PCI and PCII to make a scatter diagram (Figure 3). The Han Chinese in Shanghai (Han-10) were also assigned to the northern group in this analysis. Principal component analysis and cluster analysis produced identical results. Although Shanghai is south of the Yangtze River, these two analyses assign this city to the northern group. Not surprisingly, only 14% of individuals in the sample have both parents from Shanghai. Shanghai is a typical immigrant city.

According to the principal component analysis, the first four components can explain 83.51% of the variance (41.61%, 20.73%, 10.62% and 10.54%, for each component respectively). In a previous study of 38 loci (130 alleles, including blood groups, HLA, red cell enzymes, serum proteins etc.) in 33 Chinese ethnic groups (106 populations), principal component analysis showed that the first four components could only explain 65.8% of the variance (30.4%, 17.2%, 12.2% and 6.0%, for each component respectively) [16]. Thus, these dermatoglyphic data can explain 17.71% more of the variance than did the genetic markers. This research demonstrates that dermatoglyphics, although a classical discipline, still shows vitality and good future prospects.

**Figure 4:**
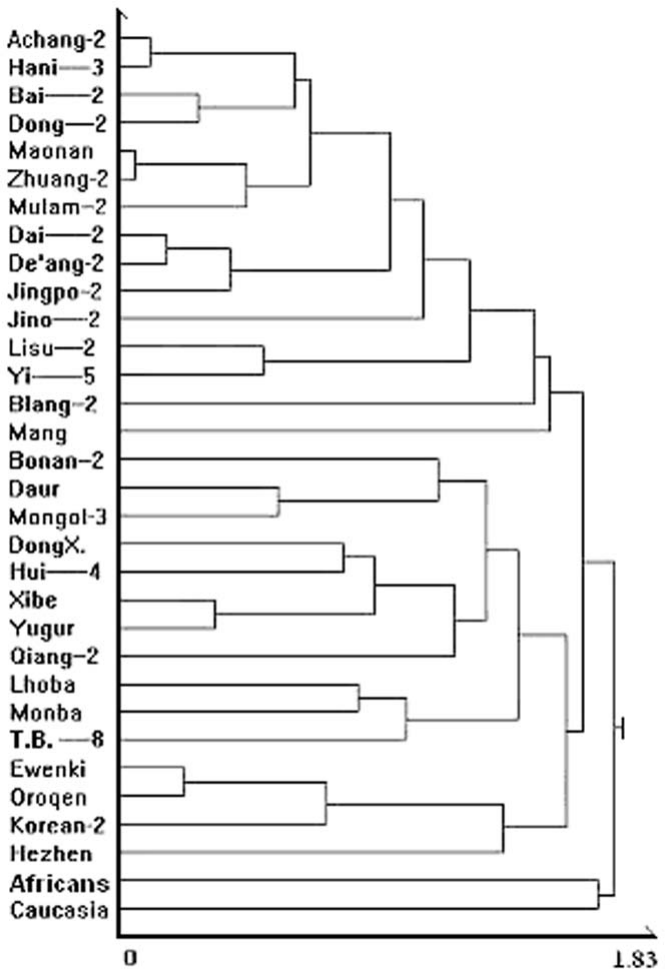
Cluster tree for Mang, 29 reference populations (PM) and 2 outgroups (SM). Mang was in the southern group. This figure was drawn based on cluster analysis using the average linkage method.

**Figure 5:**
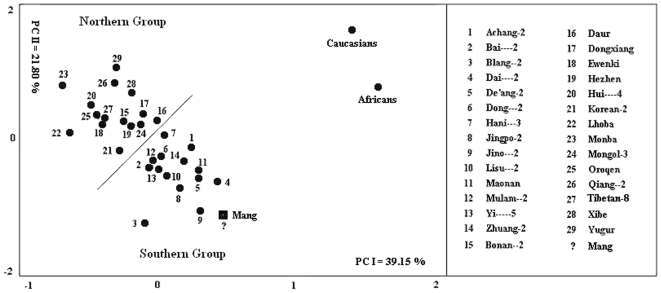
Scatter gram for principal component analysis of Mang, 29 reference populations (PM) and 2 outgroups (SM). This figure was drawn based on standardized principal component scores. Mang (indicated by the “?”) stays in the Southern group.

The Mang are a population that have not been assigned to any of the 56 Chinese ethnic groups. Therefore, we conducted a cluster analysis to determine its most closely related group. Figure 4 shows a cluster tree that includes the 31 reference samples and the Mang. The results show that the Mang cluster with the Southern Group. We also conducted principal component analysis on the 32 samples, and used PCI and PCII to make a scatter diagram (Figure 5). The Mang are also assigned to the Southern Group in this analysis. This result fits with the fact that they currently reside in southern China.

Dermatoglyphic data, coupled with cluster analysis and principal component analysis, are a useful tool for assigning Chinese populations to the northern or southern group. Dermatoglyphic data from Chinese ethnic groups can also be used as reference populations or outgroups when doing anthropological research.

## Materials and Methods

Some of the dermatoglyphic data used in this study were obtained from previously published articles or books. The authors of this paper studied 29 ethnic groups (33 samples) [6]–[10], [19], [22]–[24], [26], which account for 51.79% (29/56) of all ethnic groups. There are 6 ethnic groups with less than 10,000 people in China, and we completed research of 4 samples among them (Monba: 7500 people, Derung: 5800 people, Tatar: 5000 people, Lhoba: 2300 people). Parents of all investigated subjects are healthy and of the same ethnic group. Three samples are used as outgroups: Africans [28], [29], Caucasians [3], and Gin Vietnamese [30]. 121 samples from 56 ethnic groups in China as shown in Table 1 contain a total of 68,846 individuals with 35,950 males and 32,896 females (excluding Indian Tibetans (T.B.-1)) [16].

The standard of technical analysis for dermatoglyphics used for this research is called the Cummins' standard or the Euro-American standard [1]–[3], because it was strongly promoted by an American, H. Cummins, but was originally suggested by F. Galton (1822–1916) and E. R. Henry (1850–1931) from the U.K. [1]. The Chinese Dermatoglyphics Association (CDA) follows this Euro-American standard. According to CDA standards, 11 dermatoglyphic variables must be included in all research: total finger ridge count (TFRC), a–b ridge count (a–b RC), percentage frequencies of the arch (A), ulnar loop (Lu), radial loop (Lr) and whorl (W), percentage frequencies of true pattern in the thenar area (T/I), second interdigital area (II), third interdigital area (III), fourth interdigital area (IV) and hypothenar area (H).

SAS software was used to perform cluster analysis (see Figure S1 in Supporting Information File S1) and principal component analysis using a 156×11 data matrix. Through the computation of these two analyses, we created a cluster tree and scatter diagram using PCI and PCII (see Figure S2 in Supporting Information File S2). We also developed some computer programs for frequency calculating or weighting using QBASIC or C++.

Dermatoglyphic data from other research teams used in this paper has been carefully checked. The total frequency for several dermatoglyphic variables must add up to 100%. If the total did not reach 100%, this could have been caused by publication error or miscalculation, and needed to be corrected. No data were included in the research when there was no way to correct for such errors.

All dermatoglyphics were obtained by ink print. All our analyses on dermatoglyphics were based on these ink prints.

No data were included in the research when there was no way to correct for such errors.

## Supporting Information

Supporting Information File S1 (0.09 MB DOC)

Supporting Information File S2 (0.61 MB DOC)
