# Supplementary material for: Dermatoglyphics from All Chinese Ethnic Groups Reveal Geographic Patterning
Source: PLoS One. 2010 Jan 20;5(1):e8783. doi: 10.1371/journal.pone.0008783 (PMC2808343; doi:10.1371/journal.pone.0008783)
Supplement: Supporting Information File S1 — (0.09 MB DOC) [file pone.0008783.s001.doc]

**《Dermatoglyphics from All Chinese Ethnic Groups Reveal Geographic Patterning》**


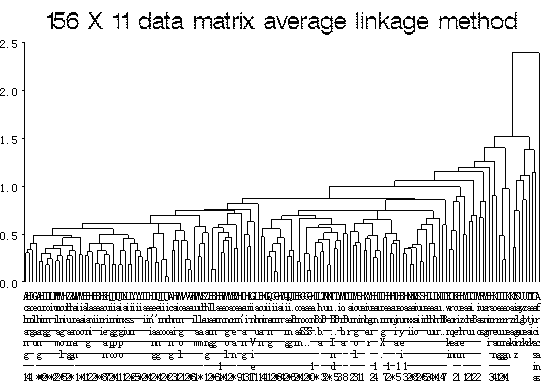


Figure S1 The cluster procedure, program and tree

**1. The Cluster Procedure**

156 X 11 data matrix average linkage method

09:06 Monday, September 27, 2009

Average Linkage Cluster Analysis

Variable Mean Std Dev Skewness Kurtosis Bimodality

x1 137.7 11.1019 0.1952 0.7436 0.2730

x2 37.3554 1.9023 -0.5899 0.9557 0.3358

x3 2.4672 1.0705 1.6260 4.7721 0.4653

x4 48.0004 4.8465 0.5891 1.1884 0.3171

x5 2.6554 0.9841 3.1908 13.9433 0.6576

x6 46.8769 5.7399 -0.5472 1.4477 0.2883

x7 5.6103 2.9104 0.6536 0.1293 0.4476

x8 1.2734 1.0938 3.3616 20.3251 0.5260

x9 15.1446 6.7987 1.8546 5.2405 0.5349

x10 67.2789 10.6963 -0.9873 2.0057 0.3899

x11 14.5562 6.1884 1.5227 3.5532 0.5019

Eigenvalues of the Correlation Matrix

Eigenvalue Difference Proportion Cumulative

1 3.29273291 1.00614205 0.2993 0.2993

3 1.19742808 0.04803918 0.1089 0.6161

4 1.14938889 0.31577408 0.1045 0.7206

5 0.83361481 0.20807186 0.0758 0.7963

6 0.62554295 0.06788945 0.0569 0.8532

7 0.55765350 0.10082872 0.0507 0.9039

8 0.45682478 0.13880402 0.0415 0.9454

9 0.31802076 0.03581829 0.0289 0.9743

10 0.28220247 0.28220247 0.0257 1.0000

11 0.00000000 0.0000 1.0000

The data have been standardized to mean 0 and variance 1

Root-Mean-Square Total-Sample Standard Deviation = 1

Root-Mean-Square Distance Between Observations = 4.690416

Cluster History

NCL --Clusters Joined--- FREQ Dist

155 Jino---2 Jino---* 2 0.0572

154 Gin----2 Gin----* 2 0.1046

153 GaoS.--2 GaoS.--* 2 0.1132

152 Blang--2 Blang--* 2 0.1287

151 Va-----2 Va-----* 2 0.132

150 Dong---2 Dong---* 2 0.1736

149 Dai----1 Jingpo-1 2 0.1739

147 Naxi---2 Naxi---* 2 0.1825

146 Hani---* Korean-3 2 0.1834

145 Lisu---2 Lisu---* 2 0.1838

144 Jingpo-2 Jingpo-* 2 0.1847

143 Bai----2 Bai----* 2 0.1953

142 De'ang-2 De'ang-* 2 0.2022

141 Bonan--2 Bonan--* 2 0.2129

140 Achang-* Hui----* 2 0.2134

139 Sui----2 Sui----* 2 0.2152

138 Hui----2 Mulam--2 2 0.2245

137 Dai----2 Dai----* 2 0.2255

136 Va-----1 CL151 3 0.2294

135 Derung-2 Derung-* 2 0.2304

134 Jino---1 CL155 3 0.2325

133 Hui----5 Zhuang-2 2 0.2375

132 CL150 Li-----* 3 0.2473

131 Han----2 Han---14 2 0.2556

130 Mulam--1 Mulam--* 2 0.2566

129 Yi-----5 Yi-----* 2 0.2597

128 Han---10 T.B.---* 2 0.2598

127 Lahu---4 Lahu---* 2 0.2621

126 Gin----1 Qiang--1 2 0.2653

125 Qiang--2 Qiang--* 2 0.2665

124 Bai----1 Blang--1 2 0.2745

123 CL133 Zhuang-* 3 0.2766

122 CL138 Mongol-* 3 0.2788

121 CL146 Hui----7 3 0.2794

120 Miao---1 Miao---* 2 0.2821

119 GaoS.--1 CL153 3 0.2882

118 Han---12 Han----* 2 0.2904

117 Bouyei Han---13 2 0.2924

116 CL140 CL132 5 0.2949

115 Han---11 Yao----* 2 0.3037

114 Achang-1 Han----4 2 0.3044

113 CL124 Hani---2 3 0.31

112 Lhoba T.B.---3 2 0.3131

111 CL126 CL154 4 0.3226

110 Achang-2 Hani---3 2 0.3256

109 Yi-----2 CL129 3 0.327

108 CL123 Maonan 4 0.3295

107 CL131 Tu 3 0.3303

106 CL149 Naxi---1 3 0.3314

105 Han----1 Xibe 2 0.3317

104 CL125 T.B.---6 3 0.3322

103 CL143 CL121 5 0.3327

102 CL114 Dong---1 3 0.3329

101 Ewenki Oroqen 2 0.3358

100 T.B.Ind. T.B.---5 2 0.345

99 CL117 CL147 4 0.3482

98 CL116 CL122 8 0.3506

97 Korean-* Man 2 0.3521

96 CL137 Hani---1 3 0.3545

95 Kazak Kirgiz 2 0.3586

94 CL99 Miao---3 5 0.3598

93 CL96 CL142 5 0.3604

92 Hui----3 Li-----1 2 0.3669

91 Li-----2 Mongol-3 2 0.3838

90 CL98 CL108 12 0.3851

89 CL94 CL139 7 0.3863

88 CL145 CL109 5 0.3989

87 CL130 She 3 0.3997

86 CL111 Han----8 5 0.4002

85 CL103 CL144 7 0.4002

84 CL118 Tujia 3 0.4004

83 CL113 CL85 10 0.4033

82 CL102 Gelao 4 0.4047

81 Lahu---3 CL127 3 0.4049

80 CL128 CL112 4 0.4105

79 CL91 Sui----1 3 0.415

78 CL100 Yi-----3 3 0.4259

77 CL110 Yi-----1 3 0.43

76 Mongol-2 CL136 4 0.4304

75 CL84 Han---15 4 0.4331

74 CL115 Mongol-1 3 0.4363

73 CL80 CL148 6 0.4411

72 CL105 Yugur 3 0.4493

71 CL106 CL88 8 0.4543

70 DongX. Han----7 2 0.4586

69 CL90 CL120 14 0.4599

68 CL86 Yi-----4 6 0.4651

67 CL74 Yao----2 4 0.4666

66 Bonan--1 Hui----4 2 0.4776

65 Derung-1 CL135 3 0.4794

64 CL72 CL107 6 0.4805

63 CL82 CL69 18 0.4824

62 De'ang-1 CL92 3 0.4866

61 Hezhen Yao----1 2 0.4886

60 CL83 CL71 18 0.4909

59 Monba T.B.---8 2 0.5042

58 CL70 CL75 6 0.5058

57 CL93 CL134 8 0.5075

56 CL87 Zhuang-1 4 0.5095

55 CL73 CL78 9 0.5101

54 Han----6 CL67 5 0.5212

53 Daur CL79 4 0.5282

52 CL77 CL76 7 0.5332

51 CL101 Korean-2 3 0.5356

50 CL89 Han----5 8 0.5444

49 CL63 CL60 36 0.5562

48 CL66 CL68 8 0.5585

47 CL119 CL55 12 0.5626

46 CL152 CL54 7 0.5691

45 Hui----6 CL56 5 0.5757

43 T.B.---4 T.B.---7 2 0.5958

42 CL47 CL59 14 0.5996

41 CL50 CL81 11 0.6021

40 CL53 CL64 10 0.6089

39 CL49 CL57 44 0.6092

38 CL39 CL52 51 0.6139

37 Lahu---2 Lisu---1 2 0.6371

36 CL62 Gin-VieT 4 0.6378

35 CL40 CL58 16 0.6469

34 CL48 CL104 11 0.6519

33 CL41 Nu-----1 12 0.6647

32 CL141 CL42 16 0.6652

31 Miao---2 Russ 2 0.6749

30 CL37 T.B.---2 3 0.6894

29 CL34 CL32 27 0.6907

28 CL38 CL45 56 0.6942

27 CL44 Han----9 10 0.7171

26 CL29 CL35 43 0.7376

25 CL95 Salar 3 0.7481

24 CL26 CL33 55 0.7493

23 CL28 CL27 66 0.7512

22 CL23 CL36 70 0.7568

21 CL22 Lahu---1 71 0.8215

20 CL51 Hui----1 4 0.8378

19 CL31 Mang 3 0.8436

18 CL24 CL43 57 0.8583

17 Uygur Uzbek 2 0.8592

16 CL21 CL18 128 0.8676

15 CL20 CL61 6 0.8938

14 Han----3 Korean-4 2 0.9047

13 CL65 Korean-1 4 0.9743

12 CL16 CL15 134 1.0004

11 CL12 CL30 137 1.0523

10 CL25 CL17 5 1.0599

9 CL10 Tatar 6 1.1169

8 Tajik Caucasia 2 1.1476

7 CL11 CL19 140 1.1551

6 CL7 Primi 141 1.1984

5 CL6 CL14 143 1.2548

4 CL9 CL8 8 1.384

3 CL5 CL13 147 1.4043

2 CL3 CL4 155 1.5185

1 CL2 Africans 156 2.3954

---------------------------------

1. **Cluster program**

title '156 X 11 data matrix average linkage method ---- Hai-guo Zhang';

**data** a;

input name $ x1-x11;

cards;

Achang-1 134.87 36.66 2.72 43.64 2.38 51.26 4.71 1.61 8.68 61.14 12.63

Achang-2 133.07 38.73 3.31 52.37 2.79 41.53 5.37 1.21 17.42 77.30 15.17

Achang-* 133.88 37.80 3.05 48.46 2.61 45.88 5.07 1.39 13.51 70.07 14.03

Bai----1 125.97 35.21 2.36 49.37 3.05 45.22 1.01 0.57 15.76 79.57 12.25

Bai----2 130.12 36.72 1.55 48.64 2.96 46.85 5.35 0.30 15.40 77.30 16.45

Bai----* 128.28 36.05 1.91 48.96 3.00 46.13 3.42 0.42 15.56 78.31 14.58

Blang--1 132.68 34.96 2.29 51.84 2.72 43.15 2.67 1.02 14.39 82.68 14.64

Blang--2 125.55 33.81 1.72 51.33 1.52 45.43 2.75 0.95 9.20 71.00 12.70

Blang--* 127.55 34.13 1.88 51.47 1.86 44.79 2.73 0.97 10.66 74.28 13.25

Bonan--1 137.96 39.21 1.06 47.89 2.89 48.16 4.73 0.00 6.57 51.81 20.50

Bonan--2 161.99 35.78 2.61 45.73 3.05 48.61 6.00 0.46 15.28 77.33 21.16

Bonan--* 156.32 36.59 2.25 46.24 3.01 48.50 5.70 0.35 13.23 71.31 21.00

Bouyei 132.99 36.68 0.85 44.80 2.25 52.10 3.24 0.89 12.83 65.86 8.93

Dai----1 130.00 38.25 2.38 47.92 2.27 47.43 1.25 0.75 13.83 72.58 10.67

Dai----2 125.37 37.50 4.00 53.68 3.18 39.14 2.78 1.54 14.35 67.87 9.63

Dai----* 127.10 37.78 3.39 51.53 2.84 42.24 2.21 1.25 14.16 69.63 10.02

Daur 144.29 37.25 2.46 44.81 3.16 49.57 3.40 1.85 24.50 57.05 17.30

De'ang-1 134.49 38.02 4.60 47.63 1.73 46.04 4.33 0.33 12.83 53.17 10.33

De'ang-2 125.33 36.79 4.16 50.59 3.47 41.78 4.83 0.42 13.31 69.75 12.46

De'ang-* 128.41 37.20 4.31 49.59 2.88 43.22 4.66 0.39 13.15 64.16 11.74

Derung-1 124.35 34.60 4.14 44.19 6.26 45.41 4.55 0.50 12.88 78.28 8.59

Derung-2 127.20 36.47 4.80 48.80 7.87 38.53 6.17 0.33 11.50 70.00 9.17

Derung-* 126.07 35.73 4.54 46.97 7.23 41.26 5.53 0.40 12.05 73.29 8.94

Dong---1 131.09 37.16 3.01 45.34 1.93 49.72 2.52 1.53 15.36 63.62 9.96

Dong---2 140.18 36.90 2.31 49.18 2.84 45.67 3.97 1.72 13.41 69.36 15.43

Dong---* 136.71 37.00 2.58 47.71 2.49 47.22 3.42 1.65 14.15 67.17 13.34

DongX. 142.88 38.02 2.29 48.50 3.18 46.03 8.81 1.74 11.75 55.03 18.58

Ewenki 147.67 36.36 2.24 44.78 2.36 50.62 6.99 1.62 7.16 25.86 19.72

GaoS.--1 162.21 40.20 1.20 38.50 2.60 57.70 8.00 0.00 14.50 79.00 14.50

GaoS.--2 163.07 39.12 1.25 40.80 2.35 55.60 9.00 0.50 17.50 68.00 11.75

GaoS.--* 162.78 39.48 1.24 40.03 2.43 56.30 8.67 0.33 16.50 71.67 12.67

Gelao 135.95 37.33 2.02 46.83 2.39 48.76 4.41 2.82 18.75 65.04 8.70

Gin----1 147.80 39.70 1.37 45.02 2.66 50.95 4.98 1.04 9.33 63.07 13.07

Gin----2 140.81 38.95 1.78 45.24 2.78 50.20 3.10 0.60 9.10 61.30 7.00

Gin----* 143.08 39.19 1.65 45.17 2.74 50.44 3.71 0.74 9.17 61.88 8.97

Han----1 151.26 39.38 2.15 43.95 2.40 51.50 5.75 3.00 20.50 70.50 19.50

Han----2 143.38 40.01 2.25 47.85 2.30 47.60 9.00 1.25 20.50 75.75 20.50

Han----3 102.40 32.34 3.71 43.61 2.32 50.36 11.28 1.13 6.02 62.97 13.16

Han----4 136.29 37.23 2.88 45.11 2.17 49.84 4.84 2.62 14.14 65.32 14.13

Han----5 135.89 39.70 2.20 44.31 1.77 51.72 7.53 1.88 14.59 68.35 5.76

Han----6 126.34 33.60 3.64 48.32 2.62 45.42 5.50 2.20 5.20 65.50 10.10

Han----7 133.25 38.09 2.60 45.41 2.39 49.60 11.41 0.42 11.96 58.32 18.79

Han----8 150.97 38.96 2.33 44.99 2.58 50.10 8.27 0.92 11.58 56.18 11.44

Han----9 127.86 31.35 2.10 47.67 2.64 47.59 4.35 3.26 21.14 77.94 11.89

Han---10 143.63 38.05 2.05 44.65 2.44 50.86 8.67 0.87 14.66 73.46 17.26

Han---11 129.87 34.07 2.06 47.14 2.06 48.74 5.00 1.69 15.92 65.00 11.65

Han---12 128.22 38.53 2.21 45.11 3.20 49.48 8.89 1.61 15.63 66.70 12.31

Han---13 131.10 36.90 0.90 43.90 2.70 52.50 3.00 2.15 14.40 68.35 11.30

Han---14 141.92 40.04 1.88 46.87 2.44 48.81 10.62 2.07 18.44 74.05 17.69

Han---15 126.77 35.77 3.45 43.65 2.54 50.36 10.14 1.69 14.72 65.95 16.33

Han----* 133.68 36.83 2.29 45.49 2.51 49.71 7.60 1.77 15.05 68.01 13.94

Hani---1 118.32 36.03 3.19 51.83 2.84 42.14 3.93 0.72 15.72 72.27 12.14

Hani---2 135.90 35.99 1.41 49.87 2.88 45.84 0.80 0.80 15.65 82.68 11.21

Hani---3 137.57 38.49 2.54 51.88 2.57 43.01 6.90 0.70 14.35 79.05 20.65

Hani---* 133.19 37.18 2.30 51.21 2.73 43.76 4.32 0.74 15.05 78.88 15.88

Hezhen 142.14 35.35 3.19 47.95 2.05 46.81 12.35 1.81 21.99 51.20 11.14

Hui----1 145.47 38.38 1.85 54.51 2.34 41.30 6.13 0.00 6.12 49.00 9.08

Hui----2 138.79 37.12 2.60 49.87 2.38 45.15 7.75 1.00 19.25 69.75 15.00

Hui----3 130.03 36.28 3.10 47.20 1.85 47.85 4.13 0.63 11.25 53.88 10.63

Hui----4 157.09 38.98 1.64 44.66 2.70 51.00 6.94 0.47 8.67 47.56 20.53

Hui----5 128.10 36.00 2.75 49.34 2.38 45.53 4.97 1.84 16.48 62.57 15.98

Hui----6 127.25 36.79 4.40 48.50 2.20 44.90 5.90 0.10 26.20 80.80 19.70

Hui----7 129.21 37.39 2.19 51.92 2.80 43.09 2.65 0.30 8.00 76.75 16.25

Hui----* 133.97 37.14 2.81 49.29 2.43 45.47 5.12 0.62 14.85 67.21 16.48

Jingpo-1 135.08 38.09 2.56 47.16 2.05 48.23 1.31 0.71 15.43 72.38 7.46

Jingpo-2 131.45 35.80 2.44 51.52 3.41 42.63 3.30 1.30 11.70 67.60 10.90

Jingpo-* 132.65 36.56 2.48 50.07 2.96 44.49 2.64 1.10 12.94 69.18 9.76

Jino---1 122.01 35.74 2.96 54.04 2.50 40.50 3.13 0.83 10.00 80.00 17.50

Jino---2 123.82 36.42 3.43 55.75 2.22 38.60 1.74 0.42 6.54 78.06 15.05

Jino---* 123.42 36.27 3.33 55.37 2.28 39.02 2.05 0.51 7.31 78.49 15.60

Kazak 134.11 37.86 2.61 52.52 4.23 40.64 9.20 2.55 30.40 61.75 34.70

Kirgiz 139.47 38.88 2.81 49.10 3.77 44.32 9.55 1.95 25.25 63.35 31.70

Korean-1 142.75 36.10 3.00 49.43 8.17 39.40 7.75 1.85 6.70 41.65 16.70

Korean-2 136.13 36.00 1.21 48.90 2.40 47.49 7.67 1.77 6.49 41.73 16.94

Korean-3 136.74 37.42 2.32 51.66 2.82 43.20 4.57 0.84 11.82 73.65 17.97

Korean-4 102.22 30.62 3.08 51.50 2.48 42.94 2.00 0.83 13.58 56.33 8.25

Korean-* 127.53 34.80 2.42 50.51 3.68 43.39 5.18 1.26 10.06 54.54 14.58

Lahu---1 141.34 35.04 1.12 57.47 2.75 38.66 5.90 1.12 8.71 80.34 19.66

Lahu---2 153.32 36.08 2.10 34.55 1.20 62.15 3.00 0.25 15.00 61.25 8.00

Lahu---3 148.61 34.92 0.95 41.90 1.69 55.46 4.94 1.06 18.35 70.90 7.14

Lahu---4 143.16 35.85 1.26 45.67 1.94 51.13 2.70 0.82 29.49 64.49 4.85

Lahu---* 145.65 35.52 1.24 44.49 1.87 52.40 3.69 0.86 22.78 67.51 7.22

Lhoba 147.05 38.40 1.47 41.72 1.54 55.27 8.58 0.15 12.95 82.53 14.31

Li-----1 133.76 36.48 2.66 48.84 2.20 46.30 2.48 0.79 5.24 42.63 12.81

Li-----2 142.88 37.08 2.87 46.04 2.90 48.19 5.29 2.96 19.00 72.67 16.22

Li-----* 138.45 36.79 2.77 47.4 2.56 47.27 3.92 1.90 12.31 58.06 14.56

Lisu---1 144.41 38.26 1.56 41.90 1.22 55.32 1.46 0.00 6.10 60.98 1.95

Lisu---2 137.56 38.33 1.98 49.95 3.83 44.24 2.17 0.57 10.92 73.95 7.92

Lisu---* 138.98 38.32 1.89 48.28 3.29 46.54 2.02 0.45 9.92 71.26 6.68

Man 126.03 33.18 2.01 49.06 2.78 46.15 6.78 0.85 8.37 51.80 16.63

Maonan 130.63 36.31 3.46 52.83 2.42 41.29 3.75 2.71 13.75 67.92 14.90

Miao---1 140.12 37.15 1.99 53.44 1.81 42.76 4.25 1.21 11.65 57.74 10.33

Miao---2 131.86 38.69 4.00 60.88 2.90 32.22 1.42 1.96 13.92 59.81 11.65

Miao---3 133.05 38.94 1.49 44.89 2.16 51.46 3.44 1.49 11.43 74.08 8.35

Miao---* 134.81 38.31 2.46 52.70 2.30 42.54 3.03 1.56 12.31 64.46 10.03

Monba 157.91 39.46 1.07 39.20 1.80 57.93 7.14 0.00 17.05 72.81 25.58

Mongol-1 123.70 32.37 2.53 46.30 2.47 48.70 2.33 1.42 15.67 58.92 14.25

Mongol-2 133.40 40.05 2.39 55.89 1.83 39.89 5.51 0.69 14.12 71.07 7.02

Mongol-3 143.34 35.97 1.84 45.53 2.83 49.80 7.51 2.33 24.47 67.01 15.02

Mongol-* 135.40 36.31 2.18 48.86 2.44 46.52 5.61 1.60 19.13 66.21 12.40

Mulam--1 126.41 36.99 4.33 48.52 2.57 44.58 7.91 1.54 16.22 85.12 14.68

Mulam--2 135.25 36.93 2.67 51.06 1.87 44.40 6.73 1.45 15.96 72.50 13.56

Mulam--* 130.97 36.96 3.47 49.83 2.21 44.49 7.30 1.49 16.09 78.60 14.10

Naxi---1 132.02 36.99 1.89 46.52 2.16 49.43 2.26 0.97 16.05 81.54 13.55

Naxi---2 132.21 37.77 1.10 43.40 2.14 53.36 5.26 0.91 19.44 70.53 12.34

Naxi---* 132.13 37.44 1.44 44.73 2.15 51.68 3.98 0.94 17.99 75.24 12.86

Nu-----1 132.50 36.93 1.74 45.79 1.82 50.65 6.16 0.36 9.05 91.31 10.14

Nu-----2 149.03 39.08 1.34 45.89 2.71 50.06 6.41 0.43 16.81 73.79 8.40

Nu-----* 144.37 38.47 1.45 45.86 2.46 50.23 6.34 0.41 14.62 78.73 8.89

Oroqen 146.34 35.83 2.41 45.86 2.19 49.54 10.65 1.01 10.91 25.20 18.36

Primi 157.84 39.27 1.65 38.08 1.42 58.85 12.96 1.35 14.14 86.53 8.59

Qiang--1 145.97 39.29 1.66 43.78 2.80 51.76 7.79 0.89 7.54 64.55 9.94

Qiang--2 164.32 40.14 2.10 48.34 2.68 46.88 10.77 1.49 18.74 63.57 11.56

Qiang--* 156.62 39.78 1.91 46.43 2.73 48.93 9.52 1.24 14.04 63.98 10.88

Russ 143.87 38.45 3.93 56.97 3.39 35.71 7.14 1.79 25.89 54.46 15.18

Salar 149.40 40.21 1.72 44.85 4.95 48.48 8.58 1.72 19.36 75.98 25.49

She 134.20 37.21 3.70 49.36 2.68 44.26 11.31 1.50 15.20 70.70 13.20

Sui----1 145.40 36.32 1.79 43.32 2.05 52.84 7.33 2.61 16.13 77.03 16.45

Sui----2 136.60 37.07 1.77 41.55 1.91 54.77 2.54 1.57 11.02 72.28 13.44

Sui----* 140.34 36.75 1.78 42.30 1.97 53.95 4.57 2.01 13.19 74.30 14.72

Tajik 134.26 39.00 6.57 47.49 2.65 43.29 4.24 3.30 28.25 50.75 26.93

Tatar 146.58 41.35 2.64 59.62 4.91 32.83 4.72 2.83 39.62 59.43 41.51

T.B.Ind. 148.10 39.82 1.48 41.98 2.08 54.46 4.18 0.49 9.11 63.92 18.59

T.B.---2 145.95 39.30 1.20 38.13 1.45 59.22 4.75 0.55 4.07 50.81 16.96

T.B.---3 148.03 39.72 1.88 41.68 2.00 54.44 7.35 0.00 18.25 75.18 14.70

T.B.---4 153.56 37.12 1.97 42.25 2.52 53.26 13.42 0.71 10.66 66.19 18.65

T.B.---5 142.31 39.11 1.97 41.45 1.72 54.86 6.93 1.07 9.49 72.60 17.91

T.B.---6 161.49 39.79 1.87 47.45 3.60 47.08 9.98 0.70 14.26 72.40 10.06

T.B.---7 168.10 34.95 3.04 44.71 3.00 49.25 11.20 1.60 6.00 63.95 12.30

T.B.---8 143.62 38.01 1.18 41.74 2.73 54.35 6.10 0.60 11.70 82.00 25.90

T.B.---* 153.00 38.01 1.92 42.92 2.57 52.59 8.44 0.82 10.31 70.03 17.08

Tu 143.47 39.66 1.92 50.98 2.90 44.20 7.95 1.64 19.16 73.36 21.96

Tujia 120.04 38.54 2.43 45.84 1.86 49.87 8.51 1.48 12.97 60.79 16.43

Uygur 138.09 37.27 2.51 50.28 3.75 43.46 14.90 4.70 39.15 62.00 33.10

Uzbek 152.00 38.00 3.46 49.39 2.76 44.39 5.91 5.63 45.67 54.38 27.00

Va-----1 137.78 37.63 2.01 56.36 2.09 39.54 2.80 0.58 16.28 77.56 9.75

Va-----2 139.60 38.20 2.34 57.61 2.82 37.23 2.67 1.06 14.39 73.67 13.67

Va-----* 138.76 37.94 2.19 57.03 2.48 38.30 2.73 0.84 15.26 75.46 11.86

Xibe 146.50 39.00 1.81 45.39 2.63 50.17 7.50 1.80 21.05 64.95 21.00

Yao----1 123.14 35.69 2.51 51.63 1.96 43.90 12.45 0.92 20.71 52.25 7.55

Yao----2 128.45 34.00 3.20 43.58 2.41 50.81 1.47 2.48 13.79 65.07 7.54

Yao----* 125.93 34.80 2.87 47.39 2.20 47.54 6.67 1.74 17.07 58.99 7.54

Yi-----1 150.63 40.38 2.12 52.50 2.76 42.62 6.18 1.18 14.27 79.27 16.91

Yi-----2 139.15 39.42 1.33 52.50 2.47 43.70 2.25 0.25 16.13 79.00 13.38

Yi-----3 135.08 37.80 1.10 43.60 1.52 53.78 4.00 0.00 12.80 67.20 17.80

Yi-----4 153.48 41.34 2.00 46.37 3.09 48.54 5.73 1.16 7.73 48.79 11.50

Yi-----5 135.38 38.90 1.62 51.20 2.82 44.36 2.00 0.20 16.15 66.60 9.50

Yi-----* 141.09 39.41 1.62 49.28 2.57 46.53 3.60 0.47 13.75 66.81 12.86

Yugur 147.40 40.71 2.03 44.30 2.29 51.38 9.05 1.63 18.99 55.79 25.07

Zhuang-1 133.40 37.79 3.98 48.20 2.00 45.82 5.50 2.60 25.00 75.60 14.30

Zhuang-2 129.55 36.27 2.75 52.09 2.63 42.53 5.30 1.70 15.10 68.50 18.50

Zhuang-* 131.35 36.98 3.32 50.27 2.34 44.07 5.39 2.12 19.73 71.82 16.54

Mang 118.42 36.71 4.10 62.44 2.35 31.11 6.41 0.00 8.98 64.10 6.62

Gin-VieT 128.00 36.30 5.40 46.90 1.70 46.00 0.40 1.10 12.30 65.20 9.30

Africans 124.72 37.62 4.85 64.70 2.70 27.75 1.13 9.40 41.75 83.50 34.13

Caucasia 131.65 41.35 7.95 61.45 4.40 26.20 7.10 2.50 37.65 45.85 35.20

;

**proc** **cluster** data=a outtree=treedat method=average std simple;

var x1-x11;

id name;

**proc** **tree** data=treedat pages=**1** space=**1**;

id name;

**run**;

title ' 080124.sas & 090927.sas ' ;

title '156 X 11 data matrix average linkage method';

===================

1. **Cluster tree**

-----end

2009, Nov., 10
