# Supplementary material for: Dermatoglyphics from All Chinese Ethnic Groups Reveal Geographic Patterning
Source: PLoS One. 2010 Jan 20;5(1):e8783. doi: 10.1371/journal.pone.0008783 (PMC2808343; doi:10.1371/journal.pone.0008783)
Supplement: Supporting Information File S2 — (0.61 MB DOC) [file pone.0008783.s002.doc]

**《Dermatoglyphics from All Chinese Ethnic Groups Reveal Geographic Patterning》**

**
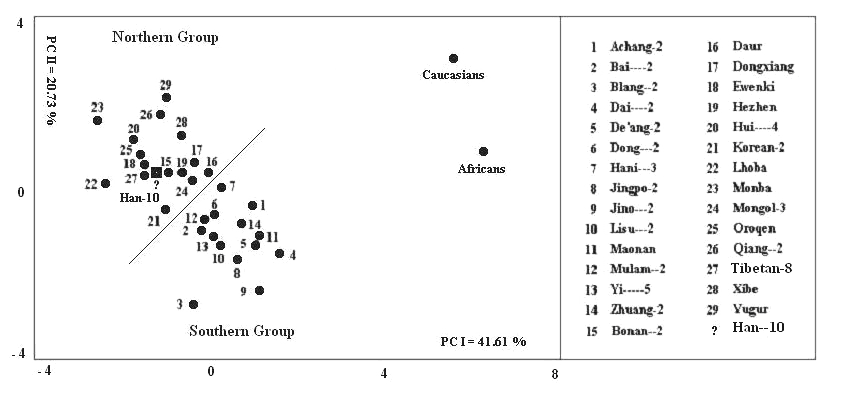
**

Figure S2 - Mathematical transformation processes for principal component analysis

The following are the mathematical transformation processes for principal component analysis of dermatoglyphics data in this research.

Principal component analysis (PCA) is a method of data reduction and is useful for dermatoglyphics research. It involves a mathematical procedure that transforms a number of possibly correlated variables into a smaller number of uncorrelated variables called principal components (PC). The first principal component accounts for as much of the variance in the data as possible, and each succeeding component accounts for as much of the remaining variance as possible.

In a general i × j data matrix, i is the number of samples, and j is the number of variables. For an i × j matrix of dermatoglyphics data, i is the number of populations of different ethnic groups, and j is the number of dermatoglyphics variables. According to Chinese Dermatoglyphics Association standards, dermatoglyphics studies must include a specific set of 11 dermatoglyphics variables. This regulation allows dermatoglyphics scholars to compare their research results with previously published dermatoglyphics data from different research teams. Therefore, for our study, *j* = 11. Since every ethnic group in China needs to be studied, *i* must be at least 56.

From the original dermatoglyphics data on populations to the scatter diagrams drawn using PCI and PCII scores to explore relationships among populations, three mathematical transformation processes were needed to perform our principal component analysis: (1) standardization of dermatoglyphics variable values, (see Table 2), (2) standardization of principal component score coefficients (see Table 6) and (3) standardization of principal component scores (see Table 7). Table 8 is correlation matrix for 31 populations and Han-10 of Shanghai of 11 variables.

**1. Standardization of original values of dermatoglyphics variables**

It is possible that the values reported for different dermatoglyphics variables can have different units or value ranges; thus, standardization of dermatoglyphics variable values is essential. Such standardization converts the mean of each variable for all samples to 0, and the standard deviation to 1.

We shall use a case from the paper to demonstrate this standardization procedure. There were 32 population samples used in this analysis, and each sample had 11 dermatoglyphics variables. Among these 32 samples, 29 were reference populations for southern and northern groups in China, two were outgroups (Africans and Caucasians), and one represented Han Chinese from Shanghai which we wanted to assign properly to either the southern or northern group. After the PCA, we placed these 32 populations on a scatter diagram using PCI and PCII scores.

Table 1 list the sample of the 31 populations and Han-10 of Shanghai and the values of the 11 dermatoglyphics variables for these populations.

The following is the standardization formula:

X_ik_ =( X_ij_ － Combini) / SD_k_；i = 1,2,…,n；k = 1,2,…,p (formula 1-1)

X_ik_ is the standardized value for the *kth* variable in the *ith* observation. X_ik_ is the original value for the *kth* variable in the *ith* observation. Combin_k_ is the mean for the *kth* variable. SD_k_ is the standard deviation for the *kth* variable.

After standardization, the mean for each variable equals 0 and its standard deviation equals 1.

These standardized values for the dermatoglyphics variables are listed in Table 2.

**Table 1 Original values of 11 dermatoglyphics variables for 31 populations and Han-10 of Shanghai**

| No. | PM^a^ & SM^b^ | Ethnic group | TFRC  x1 | a-bRC  x2 | A  x3 | Lu  x4 | Lr  x5 | W  x6 | T/I  x7 | II  x8 | III  x9 | IV  x10 | H  x11 |
| --- | --- | --- | --- | --- | --- | --- | --- | --- | --- | --- | --- | --- | --- |
| 1 | PM-S^c^ | Achang-2 | 133.07 | 38.73 | 3.31 | 52.37 | 2.79 | 41.53 | 5.37 | 1.21 | 17.42 | 77.30 | 15.17 |
| 2 | PM-S | Bai----2 | 130.12 | 36.72 | 1.55 | 48.64 | 2.96 | 46.85 | 5.35 | 0.30 | 15.40 | 77.30 | 16.45 |
| 3 | PM-S | Blang--2 | 125.55 | 33.81 | 1.72 | 51.33 | 1.52 | 45.43 | 2.75 | 0.95 | 9.20 | 71.00 | 12.70 |
| 4 | PM-N^d^ | Bonan--2 | 161.99 | 35.78 | 2.61 | 45.73 | 3.05 | 48.61 | 6.00 | 0.46 | 15.28 | 77.33 | 21.16 |
| 5 | PM-S | Dai----2 | 125.37 | 37.50 | 4.00 | 53.68 | 3.18 | 39.14 | 2.78 | 1.54 | 14.35 | 67.87 | 9.63 |
| 6 | PM-N | Daur | 144.29 | 37.25 | 2.46 | 44.81 | 3.16 | 49.57 | 3.40 | 1.85 | 24.50 | 57.05 | 17.30 |
| 7 | PM-S | De'ang-2 | 125.33 | 36.79 | 4.16 | 50.59 | 3.47 | 41.78 | 4.83 | 0.42 | 13.31 | 69.75 | 12.46 |
| 8 | PM-S | Dong---2 | 140.18 | 36.90 | 2.31 | 49.18 | 2.84 | 45.67 | 3.97 | 1.72 | 13.41 | 69.36 | 15.43 |
| 9 | PM-N | DongX. | 142.88 | 38.02 | 2.29 | 48.50 | 3.18 | 46.03 | 8.81 | 1.74 | 11.75 | 55.03 | 18.58 |
| 10 | PM-N | Ewenki | 147.67 | 36.36 | 2.24 | 44.78 | 2.36 | 50.62 | 6.99 | 1.62 | 7.16 | 25.86 | 19.72 |
| 11 | PM-S | Hani---3 | 137.57 | 38.49 | 2.54 | 51.88 | 2.57 | 43.01 | 6.90 | 0.70 | 14.35 | 79.05 | 20.65 |
| 12 | PM-N | Hezhen | 142.14 | 35.35 | 3.19 | 47.95 | 2.05 | 46.81 | 12.35 | 1.81 | 21.99 | 51.20 | 11.14 |
| 13 | PM-N | Hui----4 | 157.09 | 38.98 | 1.64 | 44.66 | 2.70 | 51.00 | 6.94 | 0.47 | 8.67 | 47.56 | 20.53 |
| 14 | PM-S | Jingpo-2 | 131.45 | 35.80 | 2.44 | 51.52 | 3.41 | 42.63 | 3.30 | 1.30 | 11.70 | 67.60 | 10.90 |
| 15 | PM-S | Jino---2 | 123.82 | 36.42 | 3.43 | 55.75 | 2.22 | 38.60 | 1.74 | 0.42 | 6.54 | 78.06 | 15.05 |
| 16 | PM-N | Korean-2 | 136.13 | 36.00 | 1.21 | 48.90 | 2.40 | 47.49 | 7.67 | 1.77 | 6.49 | 41.73 | 16.94 |
| 17 | PM-N | Lhoba | 147.05 | 38.40 | 1.47 | 41.72 | 1.54 | 55.27 | 8.58 | 0.15 | 12.95 | 82.53 | 14.31 |
| 18 | PM-S | Lisu---2 | 137.56 | 38.33 | 1.98 | 49.95 | 3.83 | 44.24 | 2.17 | 0.57 | 10.92 | 73.95 | 7.92 |
| 19 | PM-S | Maonan | 130.63 | 36.31 | 3.46 | 52.83 | 2.42 | 41.29 | 3.75 | 2.71 | 13.75 | 67.92 | 14.90 |
| 20 | PM-N | Monba | 157.91 | 39.46 | 1.07 | 39.20 | 1.80 | 57.93 | 7.14 | 0.00 | 17.05 | 72.81 | 25.58 |
| 21 | PM-N | Mongol-3 | 143.34 | 35.97 | 1.84 | 45.53 | 2.83 | 49.80 | 7.51 | 2.33 | 24.47 | 67.01 | 15.02 |
| 22 | PM-S | Mulam--2 | 135.25 | 36.93 | 2.67 | 51.06 | 1.87 | 44.40 | 6.73 | 1.45 | 15.96 | 72.50 | 13.56 |
| 23 | PM-N | Oroqen | 146.34 | 35.83 | 2.41 | 45.86 | 2.19 | 49.54 | 10.65 | 1.01 | 10.91 | 25.20 | 18.36 |
| 24 | PM-N | Qiang--2 | 164.32 | 40.14 | 2.10 | 48.34 | 2.68 | 46.88 | 10.77 | 1.49 | 18.74 | 63.57 | 11.56 |
| 25 | PM-N | T.B.---8 | 143.62 | 38.01 | 1.18 | 41.74 | 2.73 | 54.35 | 6.10 | 0.60 | 11.70 | 82.00 | 25.90 |
| 26 | PM-N | Xibe | 146.50 | 39.00 | 1.81 | 45.39 | 2.63 | 50.17 | 7.50 | 1.80 | 21.05 | 64.95 | 21.00 |
| 27 | PM-S | Yi-----5 | 135.38 | 38.90 | 1.62 | 51.20 | 2.82 | 44.36 | 2.00 | 0.20 | 16.15 | 66.60 | 9.50 |
| 28 | PM-N | Yugur | 147.40 | 40.71 | 2.03 | 44.30 | 2.29 | 51.38 | 9.05 | 1.63 | 18.99 | 55.79 | 25.07 |
| 29 | PM-S | Zhuang-2 | 129.55 | 36.27 | 2.75 | 52.09 | 2.63 | 42.53 | 5.30 | 1.70 | 15.10 | 68.50 | 18.50 |
| 30 | SM-Af | Africans | 124.72 | 37.62 | 4.85 | 64.70 | 2.70 | 27.75 | 1.13 | 9.40 | 41.75 | 83.50 | 34.13 |
| 31 | SM-Ca | Caucasia | 131.65 | 41.35 | 7.95 | 61.45 | 4.40 | 26.20 | 7.10 | 2.50 | 37.65 | 45.85 | 35.20 |
| 32 | **?**^e^ | ? Han-10 | 143.63 | 38.05 | 2.05 | 44.65 | 2.44 | 50.86 | 8.67 | 0.87 | 14.66 | 73.46 | 17.26 |
| Combin_k_ | | | 139.67 | 37.51 | 2.57 | 49.07 | 2.68 | 45.68 | 6.04 | 1.46 | 16.04 | 64.97 | 17.55 |
| SD_k_ | | | 10.91 | 1.66 | 1.33 | 5.33 | 0.62 | 6.70 | 2.83 | 1.62 | 7.74 | 14.94 | 6.42 |

a: PM-population marker.

b: SM-supervisory marker.

c: N- northern population.

d: S-southern population.

e: ?-simulated population remaining to be determined (Shanghai，han-10).

**Table 2 Standardized values of 11 dermatoglyphics variables for 31 populations and Han-10 of Shanghai**

| No. | PM & SM | TFRC  x1 | a-bRC  x2 | A  x3 | Lu  x4 | Lr  x5 | W  x6 | T/I  x7 | II  x8 | III  x9 | IV  x10 | H  x11 |
| --- | --- | --- | --- | --- | --- | --- | --- | --- | --- | --- | --- | --- |
| 1 | PM-S | -0.61 | 0.74 | 0.56 | 0.62 | 0.18 | -0.62 | -0.24 | -0.15 | 0.18 | 0.83 | -0.37 |
| 2 | PM-S | -0.88 | -0.47 | -0.77 | -0.08 | 0.46 | 0.17 | -0.24 | -0.72 | -0.08 | 0.83 | -0.17 |
| 3 | PM-S | -1.29 | -2.22 | -0.64 | 0.42 | -1.87 | -0.04 | -1.16 | -0.31 | -0.88 | 0.40 | -0.76 |
| 4 | PM-N | 2.05 | -1.04 | 0.03 | -0.63 | 0.60 | 0.44 | -0.01 | -0.62 | -0.10 | 0.83 | 0.56 |
| 5 | PM-S | -1.31 | -0.00 | 1.08 | 0.86 | 0.81 | -0.98 | -1.15 | 0.05 | -0.22 | 0.19 | -1.23 |
| 6 | PM-N | 0.42 | -0.15 | -0.09 | -0.80 | 0.78 | 0.58 | -0.93 | 0.24 | 1.09 | -0.53 | -0.04 |
| 7 | PM-S | -1.31 | -0.43 | 1.20 | 0.28 | 1.28 | -0.58 | -0.43 | -0.64 | -0.35 | 0.32 | -0.79 |
| 8 | PM-S | 0.05 | -0.36 | -0.20 | 0.02 | 0.26 | -0.00 | -0.73 | 0.16 | -0.34 | 0.29 | -0.33 |
| 9 | PM-N | 0.29 | 0.31 | -0.21 | -0.11 | 0.81 | 0.05 | 0.98 | 0.17 | -0.55 | -0.67 | 0.16 |
| 10 | PM-N | 0.73 | -0.69 | -0.25 | -0.80 | -0.51 | 0.74 | 0.34 | 0.10 | -1.15 | -2.62 | 0.34 |
| 11 | PM-S | -0.19 | 0.59 | -0.02 | 0.53 | -0.17 | -0.40 | 0.30 | -0.47 | -0.22 | 0.94 | 0.48 |
| 12 | PM-N | 0.23 | -1.29 | 0.47 | -0.21 | -1.01 | 0.17 | 2.23 | 0.22 | 0.77 | -0.92 | -1.00 |
| 13 | PM-N | 1.60 | 0.89 | -0.70 | -0.83 | 0.04 | 0.79 | 0.32 | -0.61 | -0.95 | -1.17 | 0.46 |
| 14 | PM-S | -0.75 | -1.02 | -0.10 | 0.46 | 1.18 | -0.46 | -0.97 | -0.10 | -0.56 | 0.18 | -1.04 |
| 15 | PM-S | -1.45 | -0.65 | 0.65 | 1.25 | -0.74 | -1.06 | -1.52 | -0.64 | -1.23 | 0.88 | -0.39 |
| 16 | PM-N | -0.32 | -0.90 | -1.03 | -0.03 | -0.45 | 0.27 | 0.58 | 0.19 | -1.23 | -1.56 | -0.09 |
| 17 | PM-N | 0.68 | 0.54 | -0.83 | -1.38 | -1.84 | 1.43 | 0.90 | -0.81 | -0.40 | 1.18 | -0.50 |
| 18 | PM-S | -0.19 | 0.50 | -0.45 | 0.16 | 1.86 | -0.21 | -1.37 | -0.55 | -0.66 | 0.60 | -1.50 |
| 19 | PM-S | -0.83 | -0.72 | 0.67 | 0.70 | -0.42 | -0.66 | -0.81 | 0.77 | -0.30 | 0.20 | -0.41 |
| 20 | PM-N | 1.67 | 1.17 | -1.13 | -1.85 | -1.42 | 1.83 | 0.39 | -0.90 | 0.13 | 0.52 | 1.25 |
| 21 | PM-N | 0.34 | -0.92 | -0.55 | -0.66 | 0.25 | 0.62 | 0.52 | 0.54 | 1.09 | 0.14 | -0.39 |
| 22 | PM-S | -0.41 | -0.35 | 0.07 | 0.37 | -1.30 | -0.19 | 0.24 | -0.01 | -0.01 | 0.50 | -0.62 |
| 23 | PM-N | 0.61 | -1.01 | -0.12 | -0.60 | -0.79 | 0.58 | 1.63 | -0.28 | -0.66 | -2.66 | 0.13 |
| 24 | PM-N | 2.26 | 1.58 | -0.36 | -0.14 | 0.01 | 0.18 | 1.67 | 0.02 | 0.35 | -0.09 | -0.93 |
| 25 | PM-N | 0.36 | 0.30 | -1.05 | -1.37 | 0.09 | 1.30 | 0.02 | -0.53 | -0.56 | 1.14 | 1.30 |
| 26 | PM-N | 0.63 | 0.90 | -0.58 | -0.69 | -0.08 | 0.67 | 0.52 | 0.21 | 0.65 | -0.00 | 0.54 |
| 27 | PM-S | -0.39 | 0.84 | -0.72 | 0.40 | 0.23 | -0.20 | -1.43 | -0.78 | 0.01 | 0.11 | -1.25 |
| 28 | PM-N | 0.71 | 1.92 | -0.41 | -0.89 | -0.63 | 0.85 | 1.06 | 0.11 | 0.38 | -0.61 | 1.17 |
| 29 | PM-S | -0.93 | -0.74 | 0.13 | 0.57 | -0.08 | -0.47 | -0.26 | 0.15 | -0.12 | 0.24 | 0.15 |
| 30 | SM-Af | -1.37 | 0.07 | 1.72 | 2.93 | 0.04 | -2.68 | -1.74 | 4.91 | 3.32 | 1.24 | 2.58 |
| 31 | SM-Ca | -0.74 | 2.31 | 4.05 | 2.32 | 2.79 | -2.91 | 0.37 | 0.64 | 2.79 | -1.28 | 2.75 |
| 32 | **?** | 0.36 | 0.33 | -0.39 | -0.83 | -0.38 | 0.77 | 0.93 | -0.36 | -0.18 | 0.57 | -0.05 |

**2. Calculation of eigenvalues and principal component scores**

The next step is to use the principal component analysis procedure in SAS on the 11 dermatoglyphics variables from 31 populations. Through this analysis, we obtained eigenvalues for each principal component and the proportion of the variance accounted for by each principal component (see Table 3). The first 4 components accounted for more than 80% of the total variance, and only PCI and PCII have an eigenvalue larger than 1. We used these two components to make scatter diagrams for the 31 populations. Table 4 lists the weighted coefficients of the 11 dermatoglyphics variables for the first 4 components.

**Table 3 Eigenvalues and proportion of variance accounted for by each principal component**

| No. | Eigenvalue (λi) | Difference | Proportion  （p_i_） | Cumulative  （C_m_） |
| --- | --- | --- | --- | --- |
| 1 | 4.57753227 | 2.29712663 | 0.4161 | 0.4161 |
| 2 | 2.28040564 | 1.11175360 | 0.2073 | 0.6234 |
| 3 | 1.16865204 | 0.00951876 | 0.1062 | 0.7297 |
| 4 | 1.15913328 | 0.62387067 | 0.1054 | 0.8351 |
| 5 | 0.53526261 | 0.05958276 | 0.0487 | 0.8837 |
| 6 | 0.47567985 | 0.13518599 | 0.0432 | 0.9270 |
| 7 | 0.34049386 | 0.12971730 | 0.0310 | 0.9579 |
| 8 | 0.21077656 | 0.03453204 | 0.0192 | 0.9771 |
| 9 | 0.17624452 | 0.10042515 | 0.0160 | 0.9931 |
| 10 | 0.07581937 | 0.07581937 | 0.0069 | 1.0000 |
| 11 | 0.00000000 |  | 0.0000 | 1.0000 |

**Table 4 Weighted coefficients of 11 variables for the first 4 principal components**

| x_i_ | PCI | PCII | PCIII | PCIV |
| --- | --- | --- | --- | --- |
| x1 | -.300405 | 0.414925 | 0.087800 | -.041427 |
| x2 | 0.056474 | 0.445435 | 0.405374 | -.365664 |
| x3 | 0.400254 | 0.123276 | -.20641 | -.173583 |
| x4 | 0.434335 | -.138665 | -.108951 | 0.012306 |
| x5 | 0.245272 | 0.079473 | 0.019680 | -.663850 |
| x6 | -.447851 | 0.078666 | 0.125840 | 0.085926 |
| x7 | -.230880 | 0.411823 | -.318635 | 0.050051 |
| x8 | 0.325762 | 0.146216 | -.061753 | 0.511660 |
| x9 | 0.317702 | 0.352469 | 0.157788 | 0.202009 |
| x10 | 0.078506 | -.237035 | 0.783636 | 0.136366 |
| x11 | 0.174539 | 0.459453 | 0.113747 | 0.250012 |

Using the data from Table 2 and Table 4, we can calculate z scores for the first 4 principal components for each population. For a certain principal component of a population, its z score is the sum of all the standardized values of the 11 variables in Table 2, multiplied by the weighted coefficient for each variable in Table 4.

$\left\{\begin{matrix} z1=(-0.3004)\times(-0.61)+0.0565\times0.74+...+0.1745\times(-0.37) \\ z2=0.4149\times(-0.61)+0.4454\times0.74+...+0.4595\times(-0.37) \end{matrix}\right.$ (formula 1-2)

The z scores for the first 4 components of the 32 populations are listed in Table 5. The scatter diagram using z scores of PCI and PCII is presented in Figure 1. Many previous researchers have drawn scatter diagrams in the same way after employing one standardization procedure. However, we will draw another scatter diagram after employing three standardization procedures.

**Table 5 Z scores for the first 4 principal components of the 31 populations and Han-10 of Shanghai**

| No. | PM & SM | PCI  z_1_ | PCII  z_2_ | PCIII  z_3_ | PCIV  z_4_ |
| --- | --- | --- | --- | --- | --- |
| 1 | S | 1.10 | -0.40 | 0.71 | -0.54 |
| 2 | S | -0.24 | -1.12 | 0.67 | -0.27 |
| 3 | S | -0.47 | -3.09 | -0.49 | 1.69 |
| 4 | N | -1.05 | 0.49 | 0.63 | -0.16 |
| 5 | S | 1.85 | -1.70 | -0.20 | -1.10 |
| 6 | N | 0.01 | 0.46 | 0.20 | -0.21 |
| 7 | S | 1.21 | -1.41 | -0.34 | -1.47 |
| 8 | S | 0.04 | -0.77 | 0.26 | -0.07 |
| 9 | N | -0.40 | 0.78 | -0.68 | -0.65 |
| 10 | N | -1.72 | 0.62 | -2.30 | 0.22 |
| 11 | S | 0.31 | 0.04 | 0.81 | -0.14 |
| 12 | N | -0.82 | 0.52 | -2.02 | 1.07 |
| 13 | N | -2.00 | 1.35 | -0.24 | -0.77 |
| 14 | S | 0.67 | -1.92 | -0.30 | -0.84 |
| 15 | S | 1.25 | -2.67 | 0.16 | -0.03 |
| 16 | N | -1.21 | -0.52 | -1.77 | 0.48 |
| 17 | N | -2.79 | 0.18 | 1.31 | 0.83 |
| 18 | S | 0.24 | -1.52 | 0.93 | -2.13 |
| 19 | S | 1.26 | -1.32 | -0.40 | 0.63 |
| 20 | N | -2.94 | 1.89 | 1.77 | 0.74 |
| 21 | N | -0.53 | 0.28 | -0.04 | 0.74 |
| 22 | S | -0.07 | -0.79 | -0.04 | 0.91 |
| 23 | N | -1.87 | 0.94 | -2.88 | 0.40 |
| 24 | N | -1.31 | 2.04 | 0.29 | -0.68 |
| 25 | N | -1.71 | 0.52 | 1.66 | 0.19 |
| 26 | N | -0.74 | 1.45 | 0.68 | 0.24 |
| 27 | S | 0.07 | -1.23 | 0.84 | -1.10 |
| 28 | N | -1.13 | 2.51 | 0.49 | 0.21 |
| 29 | S | 0.84 | -0.94 | -0.28 | 0.41 |
| 30 | SM-Af | 7.19 | 1.13 | 0.94 | 3.43 |
| 31 | SM-Ca | 6.36 | 3.69 | -0.94 | -2.16 |
| 32 | ？Han-10 | -1.40 | 0.50 | 0.56 | 0.13 |

**Figure 1 Scatter diagram using z scores for PCI and PCII (X axis for PCI, Y axis for PCII) according to table 5**

**
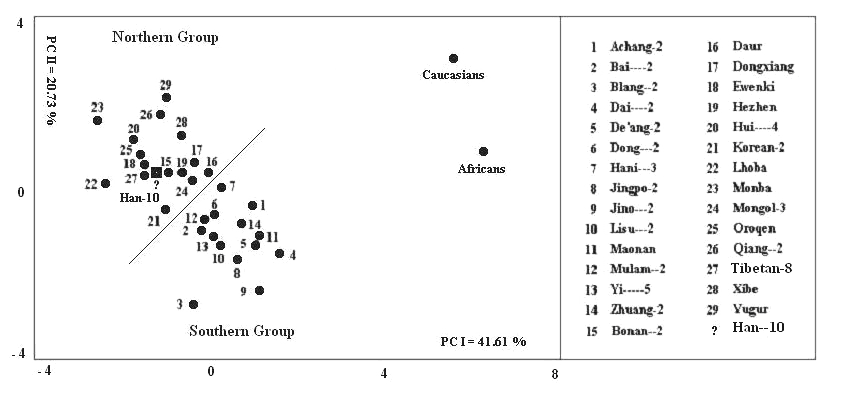
**

**3. Calculation of standardized principal component score coefficients (b_ij_)**

Although the mean of z1 and z2 is 0 in Table 5, their SD is not equal to 1. Therefore, further standardization was necessary as indicated in formula1-3.

b_ij_ = a_ij_ /λ_i_ (formula 1-3)

a_ij_ is from Table 4. λ_i_ is from Table 3. b_ij_ is the standardized principal component score coefficient of the *jth* variable of the *ith* principal component.

The following are examples of the calculations used:

The standardized principal component score coefficient of the first variable for the first component is equal to -0.0656 (= -0.3004 / 4.5775)

The standardized principal component score coefficient of the second variable for the first component is equal to 0.0123 (= 0.0565 / 4.5775)

The standardized principal component score coefficients of the 11 variables for the first 4 principal components are listed in Table 6.

**Table 6 Standardized principal component score coefficients of 11 variables for the first 4 principal components**

| No. | PCI  b_i1_ | PCII  b_i2_ | PCIII  b_i3_ | PCIV  b_i4_ |
| --- | --- | --- | --- | --- |
| 1 | -0.0656 | 0.1820 | 0.0751 | -0.0357 |
| 2 | 0.0123 | 0.1953 | 0.3469 | -0.3155 |
| 3 | 0.0874 | 0.0541 | -0.1766 | -0.1498 |
| 4 | 0.0949 | -0.0608 | -0.0932 | 0.0106 |
| 5 | 0.0536 | 0.0349 | 0.0168 | -0.5727 |
| 6 | -0.0978 | 0.0345 | 0.1077 | 0.0741 |
| 7 | -0.0504 | 0.1806 | -0.2727 | 0.0432 |
| 8 | 0.0712 | 0.0641 | -0.0528 | 0.4414 |
| 9 | 0.0694 | 0.1546 | 0.1350 | 0.1743 |
| 10 | 0.0172 | -0.1039 | 0.6705 | 0.1176 |
| 11 | 0.0381 | 0.2015 | 0.0973 | 0.2157 |

**4. Calculation of standardized principal component scores**

Using the data from Table 2 and Table 6, we can calculate standardized principal component scores for the first 4 principal components of each population. For a certain principal component of a population, its standardized z score is the sum of all the standardized values of the 11 variables in Table 2 multiplied by the standardized principal component score coefficient in Table 6.

$\left\{\begin{matrix} z1=(-0.0656)\times(-0.61)+0.0123\times0.74+...+0.0381\times(-0.37) \\ z2=0.1820\times(-0.61)+0.1953\times0.74+...+0.2015\times(-0.37) \end{matrix}\right.$ (formula 1-4)

Standard principal component scores for the 32 populations are listed in Table 7.

**Table 7 Standard principal component scores for 31 populations and Han-10 of Shanghai**

| No. | PM & SM | Ethnic groups | PCI  z_i1_ | PCII  z_i2_ | PCIII  z_i3_ | PCIV  z_i4_ |
| --- | --- | --- | --- | --- | --- | --- |
| 1 | PM-S | Achang-2 | 0.2399 | -0.1747 | 0.6042 | -0.4677 |
| 2 | PM-S | Bai----2 | -0.0529 | -0.4894 | 0.5706 | -0.2354 |
| 3 | PM-S | Blang--2 | -0.1020 | -1.3572 | -0.4173 | 1.4570 |
| 4 | PM-N | Bonan--2 | -0.2290 | 0.2155 | 0.5376 | -0.1416 |
| 5 | PM-S | Dai----2 | 0.4040 | -0.7465 | -0.1697 | -0.9512 |
| 6 | PM-N | Daur | 0.0015 | 0.2013 | 0.1736 | -0.1806 |
| 7 | PM-S | De'ang-2 | 0.2649 | -0.6173 | -0.2867 | -1.2678 |
| 8 | PM-S | Dong---2 | 0.0086 | -0.3368 | 0.2246 | -0.0644 |
| 9 | PM-N | Dongxiang | -0.0867 | 0.3428 | -0.5854 | -0.5603 |
| 10 | PM-N | Ewenki | -0.3760 | 0.2720 | -1.9678 | 0.1914 |
| 11 | PM-S | Hani---3 | 0.0682 | 0.0177 | 0.6914 | -0.1180 |
| 12 | PM-N | Hezhen | -0.1786 | 0.2268 | -1.7250 | 0.9234 |
| 13 | PM-N | Hui----4 | -0.4375 | 0.5904 | -0.2046 | -0.6614 |
| 14 | PM-S | Jingpo-2 | 0.1461 | -0.8400 | -0.2555 | -0.7281 |
| 15 | PM-S | Jino---2 | 0.2725 | -1.1702 | 0.1397 | -0.0252 |
| 16 | PM-N | Korean-2 | -0.2645 | -0.2273 | -1.5184 | 0.4181 |
| 17 | PM-N | Lhoba | -0.6095 | 0.0769 | 1.1188 | 0.7162 |
| 18 | PM-S | Lisu---2 | 0.0533 | -0.6645 | 0.7987 | -1.8339 |
| 19 | PM-S | Maonan | 0.2757 | -0.5810 | -0.3408 | 0.5417 |
| 20 | PM-N | Monba | -0.6430 | 0.8287 | 1.5115 | 0.6402 |
| 21 | PM-N | Mongol-3 | -0.1168 | 0.1244 | -0.0349 | 0.6394 |
| 22 | PM-S | Mulam--2 | -0.0156 | -0.3476 | -0.0309 | 0.7808 |
| 23 | PM-N | Oroqen | -0.4078 | 0.4133 | -2.4687 | 0.3475 |
| 24 | PM-N | Qiang--2 | -0.2862 | 0.8945 | 0.2502 | -0.5886 |
| 25 | PM-N | Tibetan-8 | -0.3730 | 0.2263 | 1.4243 | 0.1645 |
| 26 | PM-N | Xibe | -0.1612 | 0.6358 | 0.5821 | 0.2102 |
| 27 | PM-S | Yi-----5 | 0.0145 | -0.5395 | 0.7167 | -0.9459 |
| 28 | PM-N | Yugur | -0.2458 | 1.1025 | 0.4154 | 0.1799 |
| 29 | PM-S | Zhuang-2 | 0.1841 | -0.4111 | -0.2357 | 0.3549 |
| 30 | SM-Af | Africans | 1.5700 | 0.4936 | 0.8020 | 2.9551 |
| 31 | SM-Ca | Caucasians | 1.3888 | 1.6194 | -0.8029 | -1.8662 |
| 32 | **?** | Han-10 | -0.3063 | 0.2212 | 0.4827 | 0.1162 |

**5. Making scatter diagrams using standard principal component scores of PCI and PCII**

After three mathematical transformations to achieve standardization, we can plot the PCI score on the X axis, and the PCII score on the Y axis to draw scatter diagrams (Figure 2) for the 31 populations using Table 7.

**Figure 2 scatter diagrams for the 31 populations using Table 7**

**
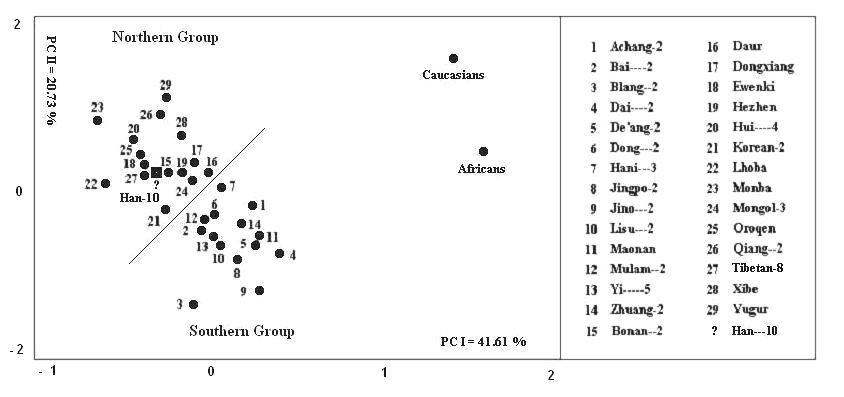
**

In Figure 2, northern ethnic groups cluster together in a group, and southern ethnic groups cluster together in another group. Africans and Caucasians separate into two groups. Shanghai Han cluster with the northern group (X = -0.3063, Y =0.2212). Shanghai Han were assigned to the northern group, which is the same result as that indicated by the cluster analysis.

**Table 8 Correlation matrix for 31 populations and Han-10 of Shanghai of 11 variables**

|  | x1 | x2 | x3 | x4 | x5 | x6 | x7 | x8 | x9 | x10 | x11 |
| --- | --- | --- | --- | --- | --- | --- | --- | --- | --- | --- | --- |
| x1 | 1.0000 | 0.3275 | -.4503 | -.7159 | -.1748 | 0.6755 | 0.6159 | -.2806 | -.0843 | -.2467 | 0.1327 |
| x2 | 0.3275 | 1.0000 | 0.1874 | -.0230 | 0.2936 | -.0459 | 0.1966 | -.0115 | 0.3673 | 0.0419 | 0.4127 |
| x3 | -.4503 | 0.1874 | 1.0000 | 0.7698 | 0.5164 | -.8589 | -.1761 | 0.4548 | 0.6054 | -.0704 | 0.3710 |
| x4 | -.7159 | -.0230 | 0.7698 | 1.0000 | 0.3818 | -.9842 | -.5024 | 0.6012 | 0.4602 | 0.1471 | 0.1602 |
| x5 | -.1748 | 0.2936 | 0.5164 | 0.3818 | 1.0000 | -.4987 | -.2782 | 0.0995 | 0.3149 | -.0436 | 0.1104 |
| x6 | 0.6755 | -.0459 | -.8589 | -.9842 | -.4987 | 1.0000 | 0.4607 | -.5780 | -.5155 | -.0991 | -.2113 |
| x7 | 0.6159 | 0.1966 | -.1761 | -.5024 | -.2782 | 0.4607 | 1.0000 | -.2076 | -.0158 | -.4518 | 0.1109 |
| x8 | -.2806 | -.0115 | 0.4548 | 0.6012 | 0.0995 | -.5780 | -.2076 | 1.0000 | 0.7020 | 0.0234 | 0.4729 |
| x9 | -.0843 | 0.3673 | 0.6054 | 0.4602 | 0.3149 | -.5155 | -.0158 | 0.7020 | 1.0000 | 0.1187 | 0.5645 |
| x10 | -.2467 | 0.0419 | -.0704 | 0.1471 | -.0436 | -.0991 | -.4518 | 0.0234 | 0.1187 | 1.0000 | -.0841 |
| x11 | 0.1327 | 0.4127 | 0.3710 | 0.1602 | 0.1104 | -.2113 | 0.1109 | 0.4729 | 0.5645 | -.0841 | 1.0000 |

******

2009-11-10
